# Warehouse-based, immunopeptidome-guided design of personalised peptide vaccines shows feasibility in clinical trial evaluation in CLL patients

**DOI:** 10.3389/fimmu.2024.1482715

**Published:** 2024-11-26

**Authors:** Jonas S. Heitmann, Susanne Jung, Marcel Wacker, Yacine Maringer, Annika Nelde, Jens Bauer, Monika Denk, Naomi Hoenisch-Gravel, Marion Richter, Melek T. Oezbek, Marissa L. Dubbelaar, Tatjana Bilich, Marina Pumptow, Peter Martus, Gerald Illerhaus, Claudio Denzlinger, Francesca Steinbach, Walter-Erich Aulitzky, Martin R. Müller, Daniela Dörfel, Hans–Georg Rammensee, Helmut R. Salih, Juliane S. Walz

**Affiliations:** ^1^ Clinical Collaboration Unit Translational Immunology, German Cancer Consortium (DKTK), Department of Internal Medicine, University Hospital Tübingen, Tübingen, Germany; ^2^ Cluster of Excellence iFIT (EXC2180) “Image-Guided and Functionally Instructed Tumor Therapies”, University of Tübingen, Tübingen, Germany; ^3^ Department of Peptide-based Immunotherapy, Institute of Immunology, University and University Hospital Tübingen, Tübingen, Germany; ^4^ German Cancer Consortium (DKTK) and German Cancer Research Center (DKFZ), partner site Tübingen, Tübingen, Germany; ^5^ Quantitative Biology Center (QBiC), University of Tübingen, Tübingen, Germany; ^6^ Institute for Clinical Epidemiology and Applied Biometry, University Hospital Tübingen, Tübingen, Germany; ^7^ Department of Hematology, Oncology and Palliative Care, Katharinenhospital Stuttgart, Stuttgart, Germany; ^8^ Department of Oncology, Hematology and Palliative Care, Marienhospital Stuttgart, Stuttgart, Germany; ^9^ Department of Hematology, Oncology and Palliative Care, Robert-Bosch-Krankenhaus, Stuttgart, Germany; ^10^ Department of Internal Medicine II, Hematology, Oncology, Clinical Immunology and Rheumatology, University Hospital Tübingen, Tübingen, Germany; ^11^ Institute of Immunology, University of Tübingen, Tübingen, Germany

**Keywords:** CLL, vaccine, peptide, warehouse, immunopeptidome, trial

## Abstract

Cancer peptide vaccination represents a promising therapeutic approach, but has been hampered by lack of suitable antigens and restricted applicability due to different HLA backgrounds of individual patients. We here introduce a novel warehouse-based concept for composition of personalized peptide vaccines and report on its successful application in a Phase II clinical trial in patients with chronic lymphocytic leukemia (CLL) after first-line therapy. 26 CLL patients in at least partial remission (PR) after 6 months of immuno-chemotherapy were vaccinated with a personalized vaccine compiled from a premanufactured peptide warehouse comprising immunopeptidome-defined CLL-associated peptides. Primary objective was evaluation of immunogenicity, secondary objectives were safety and minimal residual disease (MRD) response. Immunopeptidome-guided vaccine composition was throughout successful, proving the feasibility of warehouse-based vaccine design. Vaccination was well tolerated, with local injection site reactions being the most common adverse event. Only few patients showed vaccine-induced T cell responses, attributable to their inability to mount strong immune responses due to immune-chemotherapy and lack of potent adjuvant formulations. Both issues are addressed within a follow-up trial (NCT04688385), combining the immunopeptidome-guided warehouse-based vaccine design reported here with a potent novel adjuvant evaluating personalized multi- peptide vaccination in CLL patients under T cell supportive BTK inhibitor therapies.

**Clinical trial registration:**

www.clinicaltrialsregister.eu, identifier NCT02802943.

## Introduction

Chronic lymphocytic leukemia (CLL), the most common of adult leukemias (1), is defined by clonal expansion of small, monomorphic B cells in the peripheral blood, but also affects bone marrow and lymphatic system (2). CLL treatment has changed dramatically with targeted therapies entering the therapeutic landscape (3), displacing immuno-chemotherapy as standard frontline treatment, yet disease relapses are still caused by residual CLL cells. Several trials were initiated aiming to eliminate this minimal residual disease (MRD) by administration of chemotherapy, antibody-based immunotherapy and small molecules already approved for CLL in various novel combinations (4–7). All these studies reported high response and remission rates but also considerable side effects, especially cytopenia and infections (5), which constitutes a significant challenge for an elderly and comorbid patient collective. With most treatment discontinuation models aiming for MRD reduction or negativity (8, 9), novel low side effect MRD elimination strategies are needed in order to maintain long-lasting remissions and to improve disease-free survival (DFS) and overall survival (OS).

The immunogenicity of CLL, documented e.g. by Graft versus Leukemia-effects after hematopoietic stem cell transplantation and spontaneous remissions after viral infection, suggests that T cell-based immunotherapy might effectively target CLL (10, 11). One promising approach is peptide-based vaccination, which represents a low side-effect immunotherapeutic option relying on specific immune recognition of tumor-associated human leucocyte antigen (HLA)-presented peptides (12, 13). However, lack of broadly and naturally-presented target antigens in this low-mutational burden malignancy (14) and the challenges to design personalized vaccines have so far limited the application in large cohorts with different HLA backgrounds (15).

Within a previous study using mass spectrometry(MS)-based immunopeptidomics, we investigated the antigenic landscape of HLA-presented peptides in CLL and characterized an antigen panel across various HLA allotypes, exclusively presented on the malignant cells. These antigens were shown to elicit immune responses in CLL patients associated with improved disease outcome, which validates their role as targets of T cell-based immune control (16, 17). For a timely clinical application of these CLL-associated antigens within personalized multi-peptide vaccines we developed a premanufactured peptide “warehouse” comprising the most frequent CLL-associated antigens for different HLA allotypes. Vaccine cocktails are composed by selecting peptides from the warehouse according to the immuopeptidome analysis of any given patients’ individual CLL cells.

Here, we report on a first-in-human, Phase II trial evaluating safety, immunogenicity and preliminary efficacy of patient-individualized peptide vaccination in CLL after (immuno-) chemotherapy-based first-line therapy, as a proof of concept trial for the feasibility of personalized warehouse-based peptide-vaccine design.

## Materials and methods

### Trial design and oversight

The multi-center Phase II trial (ClinicalTrials.gov Identifier: NCT02802943) was conducted at the Clinical Collaboration Unit (CCU) Translational Immunology, University Hospital Tübingen, and four other German sites detailed in the **Supplementary Information**.

**Figure 1:**
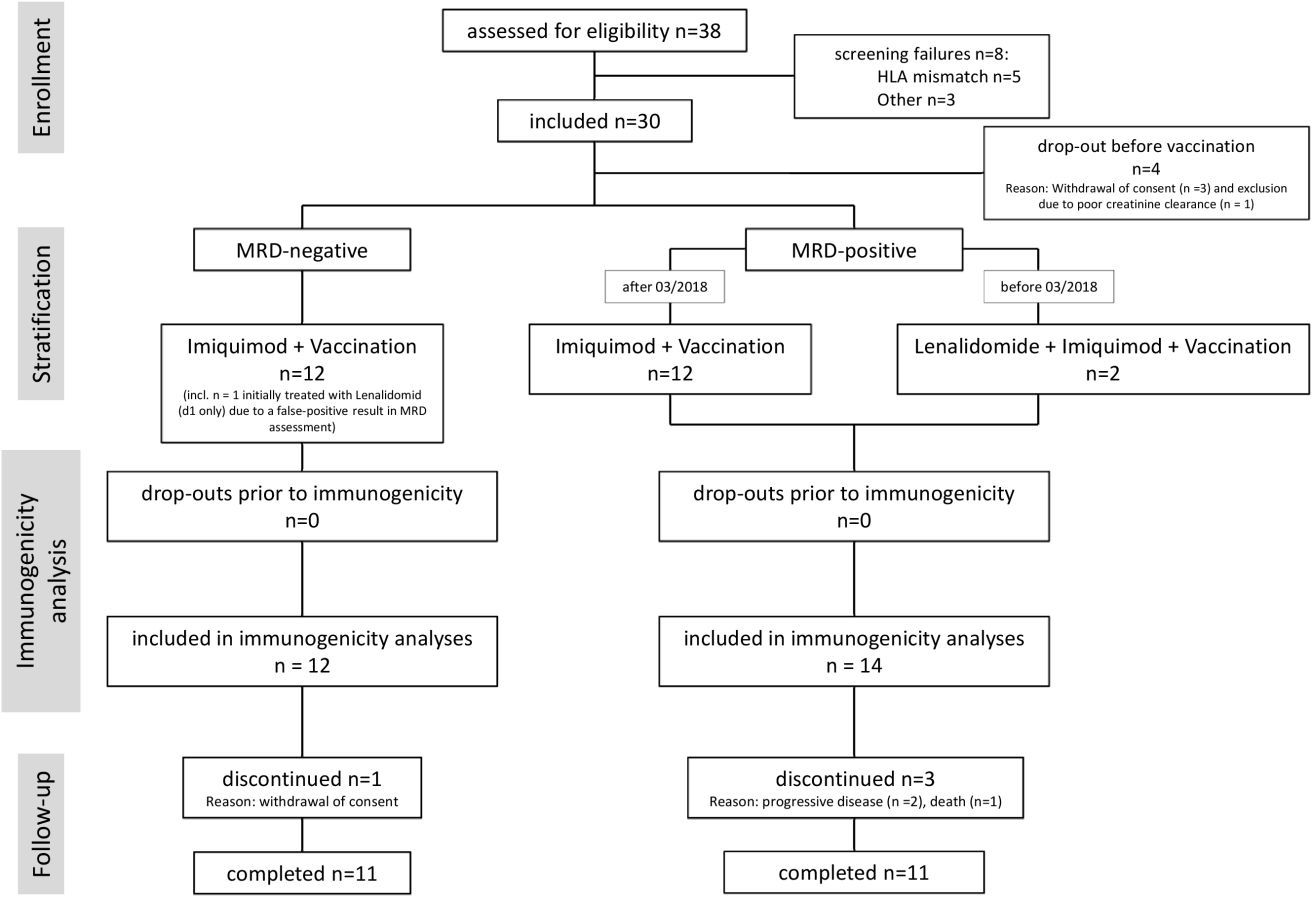
Consort flow diagram of the trial. 8 patients did not meet the inclusion criteria at screening and accordingly were not enrolled in the trial. 30 patients were enrolled in the trial, of which 4 dropped out prior to vaccination. Overall 26 patients received at least one dose of the personalized vaccine. Prior to vaccination 12 patients were minimal residual disease (MRD) negative and 14 patients MRD positive, of those latter three received lenalidomide as immune stimulator. All patients of the MRD positive arm could be included in the immunogenicity analysis, 1 patient in the MRD negative arm dropped out prior to immunogenicity analysis. All enrolled patients were assessable for safety. n, number.

Eligible patients presented with documented diagnosis of previously untreated CLL according to the International Workshop on CLL (iwCLL) guidelines, were aged ≥ 18 years and had an Eastern Cooperative Oncology Group (ECOG) performance status of 0-2. The ability to mount an immune response (analyzed by 12-day *in vitro* expansion (IVE) interferon gamma (IFN-γ) enzyme-linked immunospot (ELISpot) assay responses to an Epstein-Barr virus (EBV)/Cytomegalovirus(CMV) peptide mix) was another main inclusion criterion for the screening phase. Prerequisite for vaccination was at least partial response (PR) after first-line therapy (treating physician’s choice). Patient HLA typing had to match the HLA alleles of peptides included in the peptide warehouse (HLA-A*01, HLA-A*02, HLA-A*03, HLA-A*24, HLA-B*07 and HLA-B*08). A detailed description of inclusion and exclusion criteria is included in the supplementary information. Health status of study patients was based on medical history, laboratory values, vital signs and physical examination at screening. With regard to patient gender, only biological sex based on self-reported assessment was considered for this trial. Prior to enrollment, all patients provided written informed consent. All patients were recorded in paper-based case report forms (CRF). Up to 56 patients were initially planned, but when the immunogenicity results of the first 26 patients yielded negative results, the trial was prematurely terminated (**Figure 1**).

After screening, patients received first-line treatment according to treating physician’s choice for six months, followed by a pre-vaccination visit to determine the interim treatment outcome. If at least PR was reached, patients entered vaccination phase, with the individual peptide vaccine applied as intradermal injection on day (d)1, d4, d8, d15, d22, then every four weeks for one year until 16 vaccinations were reached (see also CLL peptide warehouse, personalized vaccine selection and adjuvant).

Initially, the trial was planned with two different arms for MRD-positive (MRD^+^) and -negative (MRD^-^) patients, with the MRD^+^ patients additionally receiving lenalidomide 2.5 mg p.o. daily as systemic immunomodulator, and combined with Aspirin^®^ (manufactured by Bayer vital GmbH, Leverkusen, Germany) (ASA) 100 mg to prevent thromboembolic events. When six CLL patients in other trials applying lenalidomide developed Philadelphia chromosome–positive B-cell acute lymphoblastic leukemia (18, 19), the lenalidomide arm was stopped, as a causal relationship of the leukemic events with the lenalidomide exposure could not be ruled out. All patients thereafter received the same treatment.

The trial was open-label without a control arm and funded by the Angewandte Klinische Forschung (AKF) program of the University Hospital Tübingen, Germany. The study was approved by the leading ethics committee of the University Hospital Tübingen (245/2016AMG1), local ethics committees at the trial sites and the Paul Ehrlich Institute (2734). The trial was registered on ClinicalTrials.gov (NCT02802943) and EudraCT number (2015-005817-61). Safety oversight was given by an independent data monitoring committee (DMC).

### CLL peptide warehouse, personalized vaccine selection and adjuvants

**Figure 2:**
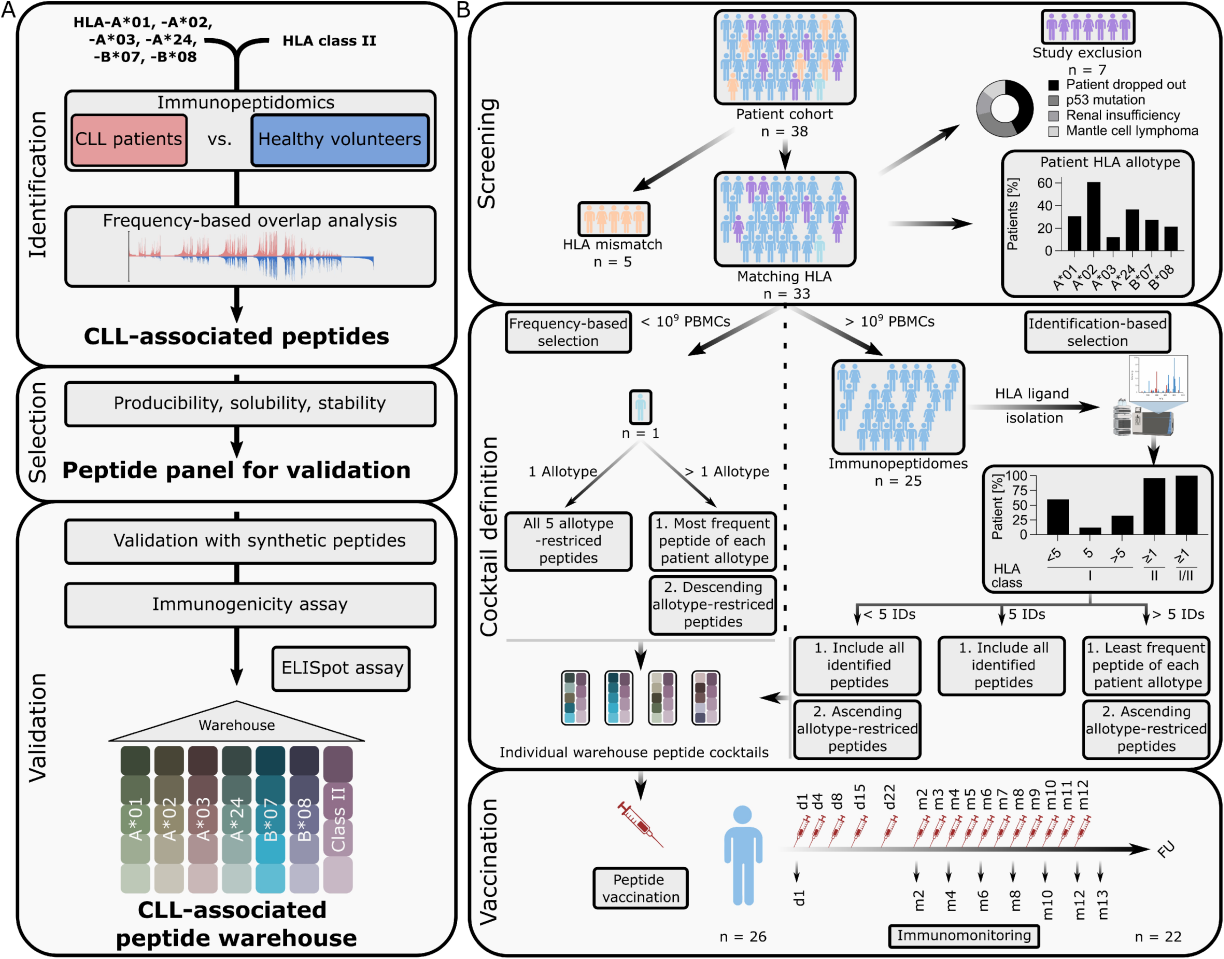
CLL-associated peptide warehouse establishment and application in clinical trial. **(A)** Chronic lymphocytic leukemia (CLL)-associated peptide warehouse establishment illustrated in a schematic workflow. A frequency-based overlap analysis was performed on peripheral blood mononuclear cells (PBMC)-derived immunopeptidomes of CLL-patients versus healthy volunteer samples. Peptide candidates were evaluated for producibility, solubility and stability and were validated by mass spectrometry (MS) using synthetic peptides. All warehouse peptides were further tested to be immunogenic. Five peptides were selected per human leukocyte antigen (HLA) class I allotype (HLA-A*01, HLA-A*02, HLA-A*03, HLA-A*24, HLA-B*07, HLA-B*08) and four peptides were selected for HLA class II. **(B)** Application of CLL-associated peptide warehouse. A CLL patient cohort (n = 38) was screened for warehouse matching HLA allotypes and other exclusion criteria as shown in the donut plot. Percentage of HLA class I allotypes of the HLA-matching patient cohort (n = 33) is shown in the upper panel (bar graph). Exclusions and corresponding exclusion criteria are shown as fraction of whole in a donut plot. Immunopeptidomes (n = 25) were analyzed and categorized in <5, 5 or >5 detected HLA class I-restricted warehouse peptides, ≥1 detected HLA class II-restricted warehouse peptides and respective length variants, or ≥1 overall warehouse peptides (HLA class I- or HLA class II-restricted including length variants (I/II)) per study patient. Vaccination took place on day (d) 1, d4, d8, d15, d22, followed by vaccinations every 4 weeks for 1 year. Immunomonitoring was performed with blood samples from d1, month (m) m2, m4, m6, m8, m10, m12, m13 and at m18 the follow-up (FU). Identifications (IDs).

The CLL peptide warehouse was developed and produced by the Good Manufacturing Practices (GMP) Peptide Laboratory at the Department of Peptide-based Immunotherapy, Institute of Immunology, University of Tübingen, Germany, and comprises 30 HLA class I-restricted CLL-associated peptides, with five peptides per HLA class I allotype (HLA-A*01, HLA-A*02, HLA-A*03, HLA-A*24, HLA-B*07, HLA-B*08) and four HLA-DR-restricted CLL-associated peptides. Warehouse peptides were found to be frequently and exclusively presented in the immmunopeptidome of CLL patients when compared to healthy peripheral blood mononuclear cell (PBMC) and B cell samples (16), and were further refined using next generation MS (**Figure 2A**). Immunogenicity of peptides was proven by *in vitro* priming of naïve T cells from healthy donors as well as detection of spontaneous peptide-specific memory T cell responses in CLL patients (**Figure 2A**). This approach delineated a set of immungenic peptides exclusivly and frequently presented on CLL cells. For the construction of a warehouse that is easy to operate, we further sorted these peptides according to HLA allotype restriction.

The 30 HLA class I warehouse peptides were restricted to the six most common HLA class I allotypes, achieving a population coverage of 92% of the European population (20); the four warehouse peptides restricted to HLA-DR showed promiscuous binding to various different HLA allotypes (further refered to as HLA class II), enabling HLA-independent application. For composition of individualized vaccine cocktails, five HLA class I-restricted peptides were selected in a personalized manner for each patient and always complemented with the same four HLA class II-restricted peptides. For HLA class I-restricted peptide selection, immunopeptidome analysis of patient PBMC samples obtained prior to the start of first-line therapy was performed. Peptides identified in the individual immunopeptidomes of the study patients’ CLL cells as part of the isolated PBMCs were preferred over undetected peptides, and if more/fewer than five peptides were identified, the peptide frequencies from the previously conducted warehouse-defining study (16) were used to select warehouse peptides in the following manner (see also **Figure 2B**, **Supplementary Table S7**):

- More than five warehouse peptides detected: one identified peptide per HLA allotype sorted by ascending peptide frequency
- Fewer than five warehouse peptides detected: all detected peptides plus patient’s HLA allotype-restricted peptides by descending frequency
- No immunopeptidome data available: most frequent peptides for each of the patient’s HLA allotype sorted by descending frequency.

Selected warehouse peptides (420 µg/peptide) were dissolved in 700 µl of 33% DMSO and applied with an injectable volume of 500 µL (corresponding to 300 µg/peptide) as intradermal injection using the dermaject^®^ intradermal injection device of Verapido Medical GmbH. 250 mg Aldara^®^ cream 5% (manufactured by MEDA Pharma GmbH & Co. KG, Bad Homburg, Germany), equivalent to 12.5 mg of the Toll-like receptor (TLR) 7 agonist imiquimod, was used as an adjuvant, was applied 18-24 h prior to vaccination on the injection site and the application area covered with plastic foil (Opsite^®^, Smith & Nephew, Watford, UK). Additionally, for MRD^+^ patients lenalidomide was planned as further adjuvant, as outlined in the previous chapter.

### Objectives and endpoints

Primary objective of the trial was to evaluate the induction of an immune response (immunogenicity) of the individually composed multi-peptide vaccine, as determined by induction of peptide-specific T cell responses at baseline (before vaccination), month (m) 2, m4, m6, m8, m10, m12, m13 and m18 by 12-d IVE IFN-γ ELISpot assays. These assays were performed after obtaining and processing the last blood sample of each individual patient at m6 and m13. Thus, the samples of each individual patient were analyzed in 2 rounds (m1-6/m7-13) to limit inter-assay variability.

Secondary objective of the trial was the evaluation of safety and toxicity of the individually composed multi-peptide vaccine. These were determined by nature, frequency, and severity of adverse events (AEs). AEs were assessed for relationship and graded by the investigator according to Common Terminology Criteria for Adverse Events (CTCAE) V4.03. Safety considerations also included clinically significant changes in laboratory values (hematology and blood chemistry) and an assessment of the vaccine composition timelines to ascertain safety and feasibility of the warehouse concept. Other secondary objectives of this trial were OS, DFS and remission status at the end of study, as well as the evaluation of MRD negativity or MRD-reduction in MRD^+^ patients.

### Blood samples

Patient PBMCs were isolated by density gradient centrifugation and kept at −80°C as short-term storage for subsequent HLA peptide isolation and T cell assays. Details on HLA peptide Isolation and analysis of HLA ligands are provided in the **Supplementary Information**.

### Immunogenicity assessment

Induction of CLL vaccine-specific T cell responses to at least one of the vaccinated peptides was evaluated at baseline (d1), m2, m4, m6, m8, m10, m12, m13, as well as at end of follow-up (FU) 18 months after first vaccination, using IFN-γ ELISpot assays after 12-day *in vitro* T cell expansion (for a detailed description please refer to **Supplementary Information**).

### Sample size calculation

Acceptance of treatment and evaluation in a phase III trial is considered worthwhile if the new treatment can produce an immune response for 35% of the analyzed patients. If the immune response rate is no better than 20%, the new treatment should be rejected from further development. These assumptions lead to a sample size of n= 56 patients with n= 28 per arm using a Simon two step minimax design (for detailed calculations please refer to supplementary information). The study was terminated early with recruitment of less than 15 patients per study arm. Therefore, the requirements for the assessment of stage 1 of the minimax-design were not met and no evaluation according to the Simon Design, i.e. no interim analysis of immune response was performed.

### Statistical analysis

The analyses include induction of peptide-specific T cell response (frequencies), overall and progression free survival (absolute and relative frequencies; median survival time including 95%-confidence intervals), the evaluation of achievement of MRD-negativity or reduction in MRD-positive patients (absolute and relative frequencies), as well as safety. Safety data are summarized by counting every respective AE (lowest level term) that occurred in a patient only once. If the same AE occurred more than once, only the highest-graded AE was counted. AEs are reported as treatment-emergent (occurring after first vaccination) and treatment-related AEs (treatment-emergent AEs, which are related to IMPs). Results are reported as frequencies, median and quartile range (Q1, Q3), box plots as median with 25% or 75% quantiles and min/max whiskers. Procedures are generally limited to descriptive analyses due to the premature termination of the study.

## Results

### Patients

From October 31, 2016 through May 20, 2020, 38 patients underwent screening, 33 patients had matching HLA allotypes, and 30 were finally enrolled in the trial. Four patients dropped out prior to first vaccination. Four patients withdrew consent at a later time point, one patient allocated to the initially planned lenalidomide arm had to be excluded due to insufficient kidney function. 26 patients were eligible for the analyses of the primary endpoint of immunogenicity. During vaccination and follow-up, two patients dropped out during vaccination and 2 after last vaccination. A total of 22 patients completed follow up. All patients who had received at least one vaccination were included in the safety and efficacy analysis (**Figure 1** Consort Flow, **Figure 2**). All 26 patients suffered from CLL according to iwCLL guidelines, with 8% stage Binet A, 54% Binet B and 38% Binet C, and a median Rai Score of 3 at screening. The age ranged from 43 to 74 years (median 64.5 years) with 11.5% of the patients being female. ECOG was 0 (53.8%) or 1 (46.2%). Prior to first vaccination, median CD4^+^ T cell counts were 136.5 (Q1, Q3: 86, 172.5), median CD8^+^ T cell counts were 151.5 (Q1, Q3: 101.5, 277). CD4^+^ and CD8^+^ T cell counts slowly increased, but were still below baseline at last FU (**Supplementary Figure S3**). A total of 61.5% of patients had received rituximab plus bendamustine, 38.5% of patients rituximab, fludarabine and cyclophosphamide as first-line therapy. No major protocol deviation occurred. At the beginning of the vaccination phase, 12 patients were MRD^-^ and 14 MRD^+^. The demographics and clinical characteristics are depicted in **Table 1**. 85% of patients, who were vaccinated at least once, completed follow-up, reasons for early termination were withdrawal of consent (n = 1), progressive disease (n = 2) and death (n = 1) (**Supplementary Table S5**).

**Table 1:** Patients’ characteristics.

Characteristics		All	MRD negative	MRD positive
Patients – n		26	12	14
Age - [years] ^†^				
	Median	64.5	60	68.5
	Range	56, 70	52, 65.5	62, 72
Sex - n (%)^†^				
	Female	3 (11.5)	2 (16.7)	1 (7.1)
	Male	23 (88.5)	10 (83.3)	13 (92.9)
Rai-Stage – n (%)^†^				
	Missing	1 (3.8)	1 (8.3)	0 (0)
	0	0 (0)	0 (0)	0 (0)
	I	0 (0)	0 (0)	0 (0)
	II	12 (46.2)	6 (50.0)	6 (42.9)
	III	3 (11.5)	1 (8.3)	2 (14.3)
	IV	10 (38.5)	4 (33.3)	6 (42.9)
Binet Stage – n (%)^†^				
	A	2 (7.7)	1 (8.3)	1 (7.1)
	B	14 (53.8)	7 (58.3)	7 (50.0)
	C	10 (38.5)	4 (33.3)	6 (42.9)
Prior treatment – n (%)^¥^				
	R-Bendamustin	16 (61.5)	6 (50)	10 (71.4)
	R-FC	10 (38.5)	6 (50)	4 (28.6)
Disease stage after prior treatment – n (%)^¥^				
	CR	13 (50)	8 (66.7)	5 (35.7)
	PR	13 (50)	4 (33.3)	9 (64.3)
MRD prior to vaccination (MRD positive only) – median (range) ^¥^	PB - CLL cell population (%)	–	–	0.1 (0.0, 0.5)
	BM - CLL cell population (%)	–	–	0.7 (0.3, 1.9)
Lymphocyte count at baseline - median (range) ^†^		87,060 (50,490, 219,200)	65,030 (29,215, 161,510)	127,340 (72,730, 273,240)
Immunstatus - %^¥^	Missing	2	1	1
	Median (range) CD4^+^	136.5 (86, 172.5)	99 (63, 153)	164 (114, 215)
	Median (range) CD8^+^	151.5 (101.5, 277)	209 (108, 272)	137 (55, 327)
Application of lenalidomide – n (%)^¥^				
	Yes	3	1*	2
	No	23	11	12
Application of at least one vaccine dose – n (%)				
	Yes	26	12	14
	No	0	0	0

Assessment was done at the time of (†) screening and at (¥) pre-vaccination visit. BM, bone marrow; R, rituximab; C, cyclophosphamide; CR, complete remission; CRi, complete remission with incomplete hematologic recovery; nPR, nodal partial remission; pB, peripheral blood; PR, partial remission; F, Fludarabine; MRD, minimal residual disease; n, number. *One patient was initially treated with lenalidomid (d1 only) due to a false-positive result in first MRD assessment.

### Vaccine production, application and validation of peptide warehouse concept for personalized vaccine design

**Figure 3:**
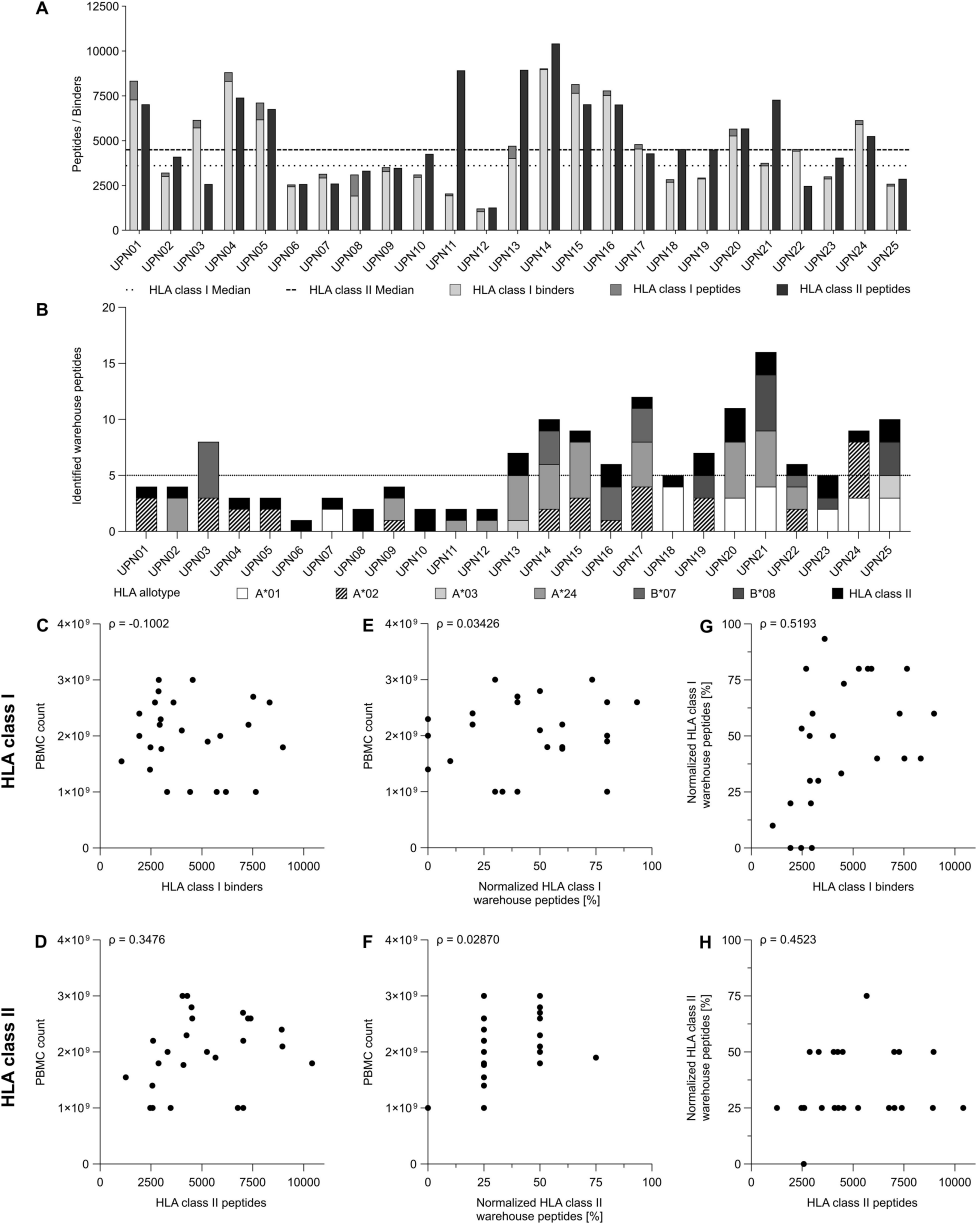
Identification and correlation analysis of study patient immunopeptidome and warehouse peptides. **(A)** Bar graph of human leukocyte (HLA) class I and HLA class II-presented peptide yields, as well as the corresponding predicted HLA class I binders on the patient’s HLA allotype superimposed with HLA class I-presented peptide yields. HLA class I binder yields are visualized as light grey, peptide yields as grey and HLA class II peptide yields as dark grey bars. Median yields are depicted as dotted (HLA class I binders) and dashed (HLA class II-restricted peptides) lines. **(B)** Bar graph of warehouse peptide identification in the respective patient’s immunopeptidome. HLA-restriction of warehouse peptides is visualized in different grey-scales and patterns. Median warehouse peptide identifications are represented as dotted line. **(C-H)** Scatter plot indicating **(C, D)** the relationship between peripheral blood mononuclear cell (PBMC) count and **(C)** HLA class I binder yields or **(D)** HLA class II-restricted peptide yields, **(E, F)** the relationship between PBMC count and **(E)** HLA allotype normalized HLA class I warehouse peptide identifications or **(F)** HLA class II warehouse peptide identifications (and respective length variants) and **(G)** HLA allotype normalized HLA class I-restricted warehouse peptide identifications and HLA class I binder yields or **(H)** HLA class II-restricted warehouse peptide identifications (and respective length variants) and HLA class II peptide yields. **(C-H)** Statistical analyses were performed using non-parametric Spearman correlation analysis. Spearman’s rho (ρ) are given in the top left of each subfigure.

Personalized warehouse-based vaccine design and production was feasible for all patients included in the trial (n = 26, **Supplementary Table S6**). Of the 33 patients with matching HLA allotypes, 10 (30%) had HLA-A*01, 20 (60%) HLA-A*02, 4 (12%) HLA-A*03, 12 (36%) HLA-A*24, 9 (27%) HLA-B*07 and 7 (21%) had HLA-B*08 (**Figure 2B**). In 25 (96%) of the 26 patients a sufficient amount of PBMCs was isolated to perform HLA ligand isolation and LC-MS/MS-based immunopeptidome analysis of patient individual CLL cells, yielding between 1050 and 8979 (median 3606) HLA class I restricted peptides and between 1261 and 10411 (median 4493) HLA class II-restricted peptides. Peptide yields did not correlate with the number of PBMC used for analysis (**Figures 3A, C, D**). Remarkably, in 25 of 25 (100%) of CLL patients’ immunopeptidomes, at least one warehouse peptide (restricted to HLA class I or HLA class II (also including length variants)) was identified, with a median of five identified peptides per patient (**Figures 2B**, **3B**; **Supplementary Table S7**). More than five HLA class I-restricted warehouse peptides were detected in eight (32%) immunopeptidomes, exactly five were identified in three (12%) immunopeptidomes and less than five were detected in 14 (56%) immunopeptidomes. For HLA class II-derived warehouse peptides, at least one of the four previously defined warehouse peptides or one of its length variants could be detected in 24 (96%) of patients-derived CLL immunopeptidomes. HLA allotype normalized warehouse-peptide detection could not be correlated with number of PBMC used for analysis (**Figures 3E, F**), however, a strong correlation between the HLA allotype normalized warehouse-peptide detection and HLA class I binder yields was observed (**Figure 3G**), and a moderate correlation was observed for HLA class II-restricted peptides and respective length variants (**Figure 3H**). Four of the 30 HLA class I-restricted warehouse peptides were never identified in the immunopeptidomes, with three of these peptides derived from the low frequency HLA-allotype HLA-A*03 (three patients with respective immunopeptidomes, **Supplementary Table S7**). Of these three peptides, all had allotype normalized peptide frequencies within the 25^th^ percentile in the previous study (16), alike the one HLA-A*01-restricted peptide. Of note, three of the four undetected warehouse peptides could be detected at least once using a highly sensitive, next-generation mass spectrometer (timsTOF) (**Supplementary Table S7**), decreasing the number of undetected warehouse peptides within the whole cohort to one (VVKPNTSSK). One HLA-A*02-derived peptide (SILEDPPSI) had a significantly increased frequency compared to a previous patient cohort and was found in 80% of immunopeptidomes (12/15 patients with matching HLA allotype) (16). This was again increased to 100% (14/14 patients in remeasurements and matching HLA allotype) using timsTOF MS. In general, allotype normalized frequency of warehouse peptide detection could be significantly increased, using timsTOF MS, while 12 of the 30 HLA class I warehouse peptides could reach a frequency of 100% (**Supplementary Figure S1**; **Supplementary Table S7**).

In sum, the very high identification rate of the warehouse peptides proves that the concept to use a immunopeptidome-guided peptide warehouse can be applied to bigger patient cohorts as well, which enables fast-track, personalized peptide-based vaccine composition and production.

### Safety

**Figure 4:**
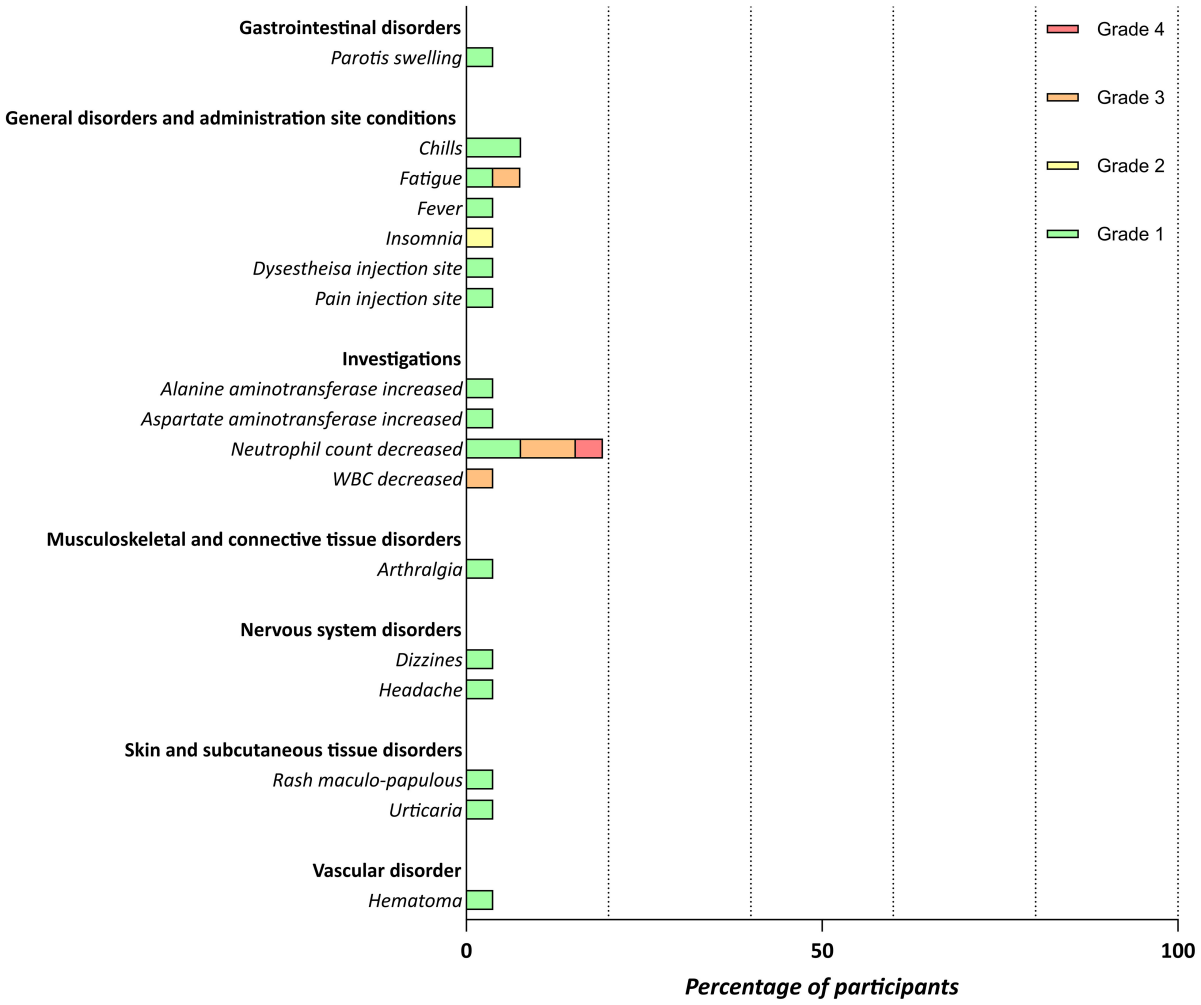
Safety of the personalized vaccination. Related adverse events (AEs) documented until last assessment. Severity was graded as mild (grade 1), moderate (grade 2), severe (grade 3), or life-threatening (grade 4) based on CTCAE V4.03.

Treatment-emergent AEs were observed in all of the 26 patients, of which 43 (18.9%) were considered related to the vaccination (**Figure 4**; **Table 2**). The most common treatment-emergent AEs of any grade in the trial were injection site reactions (n = 20), a decrease in lymphocyte count (n = 13), flu like symptoms (n = 11) and decrease in neutrophil count (n = 10). Most common treatment-related AEs of any grade were also injection site reaction (n = 20) and decrease in neutrophil count (n = 5), as well as chills (n = 2) and fatigue (n = 2). In one case, grade four toxicity with probable relation to treatment was observed (neutrophil count decrease). Overall, no serious AEs (SAEs) related to the vaccination were observed. During trial conduct, 16 (3.9% of 407 AEs reported in total) SAEs were reported for nine patients (34.6% of all the 26 patients): two patients treated in the subsequently closed lenalidomide arm (pleural effusion, coronary heart disease, acute coronary syndrome, and optic nerve disorders left and right), three patients in the MRD^+^ arm (coronary heart disease, colonic perforation, ileostoma closure, and lower leg fracture) and four patients in the MRD^-^ arm (bihilar lymphadenopathy, diarrhea, sepsis, febrile neutropenia, hyperparathyroidism, and sudden death (NOS)). There was a similar safety profile between patients with and without evidence of MRD with regard to both treatment emergent and treatment related AEs (**Supplementary Table S1**, **S2**). One patient died at m7 during the vaccination period due to an unrelated cause (term: general deterioration). No patient discontinued study treatment because of AEs related to the vaccination. No treatment-related death was observed.

**Table 2:** Treatment-emergent adverse events all patients.

SOC	CTCAE term	All subjects (n=26)					
		Grade 1	Grade 2	Grade 3	Grade 4	Grade 5	Any grade
Patients with events	All terms	127	50	41	10	1	229
Blood and lymphatic system disorders	Anemia, n (%)	2 (7.7%)	-	1 (3.8%)	-	-	3 (11.5%)
	Febrile neutropenia, n (%)	-	-	1 (3.8%)^†^	-	-	1 (3.8%)
Cardiac disorder	Bradycardia, n (%)	1 (3.8%)	-	-	-	-	1 (3.8%)
	Acute coronary syndrome, n (%)	-	-	1 (3.8%)^†^	-	-	1 (3.8%)
	Aortic valve disease, n (%)	-	-	1 (3.8%)	-	-	1 (3.8%)
	Other, coronary heart disease, n (%)	-	-	2 (7.7%)^†^	-	-	2 (7.7%)
	Sudden death (NOS), n (%)	-	-	-	-	1 (3.8%)^†^	-
Ear and labyrinth disorders	Other, sudden hearing loss, n (%)	-	1 (3.8%)	-	-	-	1 (3.8%)
	Tinnitus, n (%)	1 (3.8%)	-	-	-	-	1 (3.8%)
Endocrine disorders	Hyperparathyreodism, n (%)	-	1 (3.8%)^†^	-	-	-	1 (3.8%)
	Hypothyroidism, n (%)	-	1 (3.8%)	-	-	-	1 (3.8%)
Eye disorders	Glaucoma, n (%)	1 (3.8%)	-	-	-	-	1 (3.8%)
	Optic nerve disorder left, n (%)	-	-	1 (3.8%)^†^	-	-	1 (3.8%)
	Optic nerve disorder right, n (%)	-	1 (3.8%)^†^	-	-	-	1 (3.8%)
	Other, homonymous hemianopsia on the right eye, n (%)	-	1 (3.8%)	-	-	-	1 (3.8%)
	Other, hordeolum left eye, n (%)	-	1 (3.8%)	-	-	-	1 (3.8%)
Gastrointestinal disorders	Abdominal pain, n (%)	1 (3.8%)	-	-	-	-	1 (3.8%)
	Colonic perforation, n (%)	-	-	-	1 (3.8%)^†^	-	1 (3.8%)
	Diarrhea, n (%)	2 (7.7%)	-	1 (3.8%)^†^	-	-	3 (11.5%)
	Gastroesophageal reflux disease, n (%)	-	1 (3.8%)	-	-	-	1 (3.8%)
	Gingival pain, n (%)	-	1 (3.8%)	-	-	-	1 (3.8%)
	Ileostoma closure, n (%)	-	-	1 (3.8%)^†^	-	-	1 (3.8%)
	Mucositis oral, n (%)	1 (3.8%)	-	-	-	-	1 (3.8%)
	Nausea, n (%)	2 (7.7%)	-	-	-	-	2 (7.7%)
	Other, lactose intolerance, n (%)	1 (3.8%)	-	-	-	-	1 (3.8%)
	Other, oral thrush, n (%)	-	1 (3.8%)	-	-	-	1 (3.8%)
	Parotis swelling, n (%)	1 (3.8%)	-	-	-	-	1 (3.8%)
	Toothache, n (%)	2 (7.7%)	-	-	-	-	2 (7.7%)
General disorders and administration site conditions	Chills, n (%)	2 (7.7%)	-	-	-	-	2 (7.7%)
	Fatigue, n (%)	3 (11.5%)	-	1 (3.8%)	-	-	4 (15.4%)
	Fever, n (%)	1 (3.8%)	-	-	-	-	1 (3.8%)
	Flu like symptoms, n (%)	8 (30.7%)	3 (11.5%)	-	-	-	11 (42.3%)
	Injection site reaction, n (%)	19 (73.1%)	1 (3.8%)	-	-	-	20 (76.9%)
	Insomnia, n (%)	1 (3.8%)	1 (3.8%)	-	-	-	2 (7.7%)
	Malaise, n (%)	1 (3.8%)	-	1 (3.8%)	-	-	2 (7.7%)
	Other, dysesthesia injection site, n (%)	1 (3.8%)	-	-	-	-	1 (3.8%)
	Other, pain injection site, n (%)	1 (3.8%)	-	-	-	-	1 (3.8%)
	Syncope, n (%)	1 (3.8%)	-	-	-	-	1 (3.8%)
Hepatobiliary disorders	Other, hepatic fibrosis, n (%)	-	1 (3.8%)	-	-	-	1 (3.8%)
Immune system disorders	Other, bihilar lymphadenopathy, n (%)	-	-	1 (3.8%)^†^	-	-	1 (3.8%)
	Other, sarcoidosis, n (%)	-	1 (3.8%)	-	-	-	1 (3.8%)
Infections and infestations	Lung infection, n (%)	-	1 (3.8%)	1 (3.8%)	-	-	2 (7.7%)
	Nail infection, n (%)	1 (3.8%)	1 (3.8%)	-	-	-	2 (7.7%)
	Other, herpes genitalis infection, n (%)	-	1 (3.8%)	-	-	-	1 (3.8%)
	Other, herpes simplex labialis, n (%)	1 (3.8%)	-	-	-	-	1 (3.8%)
	Other, infection nose/throat, n (%)	1 (3.8%)	-	-	-	-	1 (3.8%)
	Other, nasolacrimal duct left eye infection, n (%)	1 (3.8%)	-	-	-	-	1 (3.8%)
	Other, shingles anal, n (%)	-	1 (3.8%)	-	-	-	1 (3.8%)
	Other, shingles, n (%)	1 (3.8%)	1 (3.8%)	-	-	-	2 (7.7%)
	Sepsis, n (%)	-	-	-	1 (3.8%)^†^	-	1 (3.8%)
	Sinusitis, n (%)	1 (3.8%)	-	-	-	-	1 (3.8%)
	Soft tissue infection right middle finger, n (%)	-	-	1 (3.8%)	-	-	1 (3.8%)
Injury, poisoning and procedural complications	Bruising, n (%)	1 (3.8%)	-	-	-	-	1 (3.8%)
	Fracture - right lower leg, n (%)	-	-	1 (3.8%)^†^	-	-	1 (3.8%)
Investigations	Alanine aminotransferase increased, n (%)	1 (3.8%)	-	1 (3.8%)	-	-	2 (7.7%)
	Aspartate, n (%)	1 (3.8%)	-	-	-	-	1 (3.8%)
	CRP increased, n (%)	1 (3.8%)	1 (3.8%)	-	-	-	2 (7.7%)
	GGT increased, n (%)	-	-	1 (3.8%)	-	-	1 (3.8%)
	Hypokalemia, n (%)	1 (3.8%)	-	-	-	-	1 (3.8%)
	LDH increased, n (%)	2 (7.7%)	-	-	-	-	2 (7.7%)
	Lymphocyte count decreased, n (%)	2 (7.7%)	-	9 (34.6%)	2 (7.7%)	-	13 (50%)
	Neutrophil count decreased, n (%)	1 (3.8%)	2 (7.7%)	4 (15.4%)	4 (15.4%)	-	11 (42.3%)
	Other, ß2 microglobulin increased, n (%)	1 (3.8%)	-	-	-	-	1 (3.8%)
	Other, hypogammaglobulinemia - IgA, n (%)	1 (3.8%)	-	-	-	-	1 (3.8%)
	Other, hypogammaglobulinemia - IgG, n (%)	3 (11.5%)	1 (3.8%)	-	-	-	4 (15.4%)
	Other, hypogammaglobulinemia - IgM, n (%)	3 (11.5%)	-	-	-	-	3 (11.5%)
	Other, IgM increased, n (%)	1 (3.8%)	-	-	-	-	1 (3.8%)
	Platelet count decreased, n (%)	4 (15.4%)	1 (3.8%)	1 (3.8%)	-	-	6 (23.1%)
	White blood cell count decreased, n (%)	2 (7.7%)	3 (11.5%)	4 (15.4%)	2 (7.7%)	-	11 (42.3%)
Metabolism and nutrition disorders	Hypercalcemia, n (%)	-	1 (3.8%)	-	-	-	1 (3.8%)
	Hyperglycemia, n (%)	-	3 (11.5%)	-	-	-	3 (11.5%)
	Hyperkalemia, n (%)	2 (7.7%)	-	-	-	-	2 (7.7%)
	Hyperuricemia, n (%)	1 (3.8%)	1 (3.8%)	1 (3.8%)	-	-	3 (11.5%)
	Hypophosphatemia, n (%)	-	-	1 (3.8%)	-	-	1 (3.8%)
Musculoskeletal and connective tissue disorders	Arthralgia, n (%)	1 (3.8%)	-	-	-	-	1 (3.8%)
	Back pain, n (%)	2 (7.7%)	-	-	-	-	2 (7.7%)
	Neck pain, n (%)	1 (3.8%)	-	-	-	-	1 (3.8%)
	Non-cardiac chest pain, n (%)	-	-	1 (3.8%)	-	-	1 (3.8%)
	Other, arthritic complaints (left toe), n (%)	-	1 (3.8%)	-	-	-	1 (3.8%)
	Other, cervical spine pain, n (%)	-	1 (3.8%)	-	-	-	1 (3.8%)
	Other, cramp in the hand, n (%)	1 (3.8%)	-	-	-	-	1 (3.8%)
	Other, cramps, n (%)	1 (3.8%)	-	-	-	-	1 (3.8%)
	Other, groin pain, n (%)	1 (3.8%)	-	-	-	-	1 (3.8%)
	Other, limb pain, n (%)	1 (3.8%)	-	-	-	-	1 (3.8%)
	Other, lumbal pain, n (%)	-	1 (3.8%)	-	-	-	1 (3.8%)
	Other, omarthalgia right, n (%)	1 (3.8%)	-	-	-	-	1 (3.8%)
	Other, pain feet, n (%)	-	1 (3.8%)	-	-	-	1 (3.8%)
	Other, pain right knee, n (%)	1 (3.8%)	-	-	-	-	1 (3.8%)
	Other, tendiniosis calcarea (left shoulder), n (%)	-	1 (3.8%)	-	-	-	1 (3.8%)
Neoplasms benign, malignant and unspecified (incl. cysts and polyps)	Other, basalioma left ear, n (%)	1 (3.8%)	-	-	-	-	1 (3.8%)
	Other, parathyroidadenoma upper left, n (%)	-	1 (3.8%)	-	-	-	1 (3.8%)
Nervous system disorders	Dizziness, n (%)	3 (11.5%)	1 (3.8%)	-	-	-	4 (15.4%)
	Headache, n (%)	1 (3.8%)	1 (3.8%)	-	-	-	2 (7.7%)
Renal and urinary disorders	Erectile dysfunction, n (%)	1 (3.8%)	-	-	-	-	1 (3.8%)
	Other, pain left kidney, n (%)	1 (3.8%)	-	-	-	-	1 (3.8%)
Respiratory, thoracic and mediastinal disorders	Cough, n (%)	3 (11.5%)	-	-	-	-	3 (11.5%)
	Epistaxis, n (%)	1 (3.8%)	-	-	-	-	1 (3.8%)
	Other, pulmonary nodule, n (%)	1 (3.8%)	-	-	-	-	1 (3.8%)
	Other, subpleural fibrosis bilateral, n (%)	1 (3.8%)	-	-	-	-	1 (3.8%)
	Pleural effusion, n (%)	-	-	1 (3.8%)^†^	-	-	1 (3.8%)
	Sore throat, n (%)	3 (11.5%)	1 (3.8%)	-	-	-	4 (15.4%)
	Upper respiratory infection, n (%)	1 (3.8%)	-	-	-	-	1 (3.8%)
Nervous system disorders	Other, lip herpes, n (%)	1 (3.8%)	-	-	-	-	1 (3.8%)
	Other, morphea, n (%)	1 (3.8%)	-	-	-	-	1 (3.8%)
	Other, redness left shin, n (%)	1 (3.8%)	-	-	-	-	1 (3.8%)
	Other, redness of the left middle finger, n (%)	1 (3.8%)	-	-	-	-	1 (3.8%)
	Other, redness right shin, n (%)	1 (3.8%)	-	-	-	-	1 (3.8%)
	Other, skin irritation perianal intermittent, n (%)	1 (3.8%)	-	-	-	-	1 (3.8%)
	Rash acneiform, n (%)	1 (3.8%)	-	-	-	-	1 (3.8%)
	Rash maculo-papular, n (%)	2 (14.3%)	-	-	-	-	2 (14.3%)
	Urticaria, n (%)	1 (3.8%)	-	-	-	-	1 (3.8%)
Surgical and medical procedures	Other, injury left hand, n (%)	-	1 (3.8%)	-	-	-	1 (3.8%)
Vascular disorders	Hematoma, n (%)	1 (3.8%)	-	-	-	-	1 (3.8%)
	Hot flashes, n (%)	1 (3.8%)	-	-	-	-	1 (3.8%)
	Hypertension, n (%)	-	6 (23.1%)	2 (7.7%)	-	-	8 (30.8%)

Adverse events (AEs) and serious AEs^†^ are classified according to CTCAE V4.03. Severity and relationship were judged by the investigator. AEs are reported until the secondary safety endpoint. For each patient, AEs occurring at least once were counted with the highest CTCAE grading. CTCAE, Common Terminology Criteria for Adverse Events; n, number; SOC, system organ class.

### Immunogenicity

**Figure 5:**
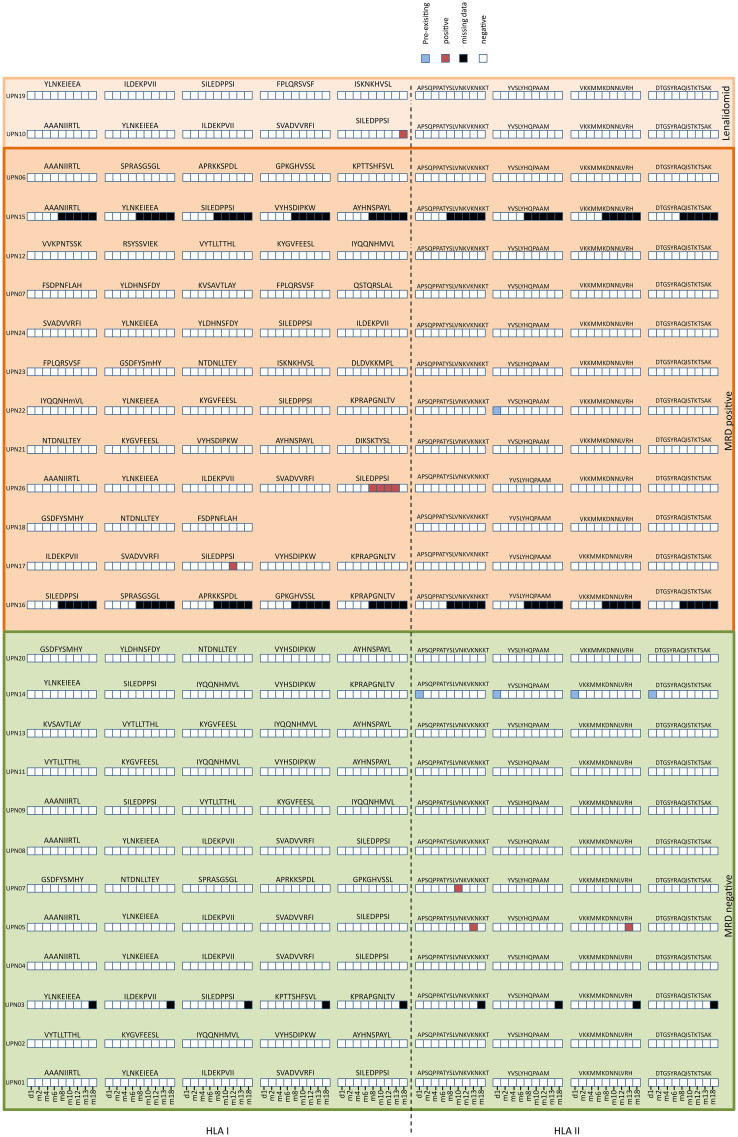
Immunogenicity of the personalized vaccination. Heatmap of personalized vaccine-induced T cell responses assessed after 12 days *in vitro* expansion by IFN-γ ELISpot assays using PBMCs from study patients collected before administration and at different time points after first administration (d1, m2, m4, m6, m8, m10, m12, m13, m18). T cell responses were assessed as pre-existing (positive prior to vaccination), positive, negative, missing data.

The immunogenicity endpoint was not reached: CLL-specific induced IFN-γ T cell responses to at least one peptide were documented only in 19.2% (5/26) of all study patients (within MRD-negative arm 16.7% (2/12), MRD positive without lenalidomide 16.7% (2/12), and MRD positive with lenalidomide 50% (1/2)) until end of study (**Figure 5**; **Supplementary Figure S2**). No statistical test for of the immune response was performed, as the requirements for the assessment of stage 1 of the minimax-design were not met,. One patient had pre-existing T cell responses for all HLA-DR peptides, which were not detectable at any consecutive time point. The earliest T cell responses after vaccination were detectable at m8. Apart from one patient, who showed T cell responses against two different HLA-DR peptides at m13 (**Supplementary Figure S2C**), patients showed T cell responses only against a single peptide. Most T cell responses were detected only once during the trial period, apart from one patient who developed a T cell response to the peptide SILEDDPSI at m8, which persisted for 5 months until the end of vaccination (**Figure 5**; **Supplementary Figure S2B**) with 34 (m8), 225 (m10), 120 (m12) and 194 (m13) spot counts per 5 x 10^5^ PBMCs.

### CLL outcome and survival

None of the patients with MRD positivity at baseline showed MRD negativity at EOT (end of treatment). MRD reduction compared to baseline was observed in n = 2 patients until EOT (**Supplementary Table S3**). Of the patients that were MRD negative at baseline, 83.3% remained MRD negative at EOT.

At FU, survival data for 25 patients were available. With the exception of one patient who had died due to an unrelated cause, all patients were still alive (**Supplementary Table S3**). During the trial, three patients had signs of progressive disease. Of note, none of the patients with a detectable T cell response (n = 5) experienced MRD conversion (from negative to positive), disease progression or death.

## Discussion

Antigen-specific immunotherapy, in particular peptide vaccination, depends on the recognition of naturally-presented antigens derived from mutated and unmutated gene products on HLA and represents a promising low-side-effect concept for cancer treatment. So far, broad application of peptide vaccines in cancer patients has been hampered by challenges of time- and cost-intensive personalized vaccine design and limited number of neoepitopes from tumor-specific mutations, especially in low-mutational burden malignancies including CLL (14). Personalized neoepitope-driven vaccine approaches are currently evaluated for various tumor entities (21–23). Targeting specific cancer mutations enables a highly tumor-specific therapy, with neoepitopes representing highly immunogenic antigen targets. Selection of mutation-derived HLA-peptides is in most cases based on genome and transcriptome sequencing of the tumor followed by different epitope prediction algorithms on patient-specific HLA allotypes (24). However, patient/tumor-specificity and intratumoral heterogeneity of somatic mutations (25), as well as the limited number of mutations that are ultimately presented as HLA-restricted neoepitopes on the tumor cells result in a rather costly process and restrict the broad applicability of neoepitopes in particular in low-mutational burden cancer entities (26). The warehouse concept in contrast enables personalized vaccine design from a preproduced peptide collection, that was identified as naturally presented, tumor-exclusive and immunogenic in the respective tumor entity by MS-based immunopeptidome analysis. This approach reduces the costs of personalized vaccine design and guarantees the natural presentation and immunogenicity of the applied antigens. However, in contrast to mutation-derived neoepitopes these self-antigens can suffer from immune tolerance, which calls for the future design of entity spanning neoepitope warehouses combining the advantages of both approaches (27).

This trial provides proof of concept for the fast-track immunopeptidome-guided design and production of a personalized multi peptide-vaccine cocktail from an established antigen warehouse for clinical use in CLL patients of various HLA backgrounds. It yielded data from 30 enrolled patients, 26 of which were eligible for analysis of immunogenicity (see **Figure 1** and **Supplementary Table S5**). Even though there was a certain drop-out rate, mostly due to retraction of consent, we do not consider this relevant for our analysis of the results regarding immunogenicity, as dropouts were before vaccination. Further dropouts during follow-up were due to progressive disease and in one case death from unrelated cause (see **Figure 2** and **Supplementary Table S5**), so we expect no impact on the study results regarding safety and toxicity either. The small sample size and premature termination of the study might bias the results, however, as the study set-up was overtaken by significant changes in the current treatment landscape of CLL (28), a prolongation and expansion would not have yielded results applicable to instigate further investigations.

MS-based immunopeptidomics represents the only unbiased method for direct identification and characterization of naturally presented tumor-peptides, a key prerequisite for the development of T cell-based vaccinations (29). Here, we have established a warehouse of premanufactured synthetic peptides for ‘off-the-shelf’ formulation, comprising unmutated HLA-presented antigens of various HLA allotypes identified by a comparative MS-based immunopeptidome approach (16, 30). We were able to show the feasibility of an immunopeptidome-guided compilation of personalized vaccines in 100% of study patients of various HLA allotypes with high identification rates of warehouse peptides within patients’ immunopeptidomes, proving warehouse-based vaccine design to be an efficient and rapid approach for further vaccination projects. Recently achieved immense technical MS advances and optimized immunopeptidomics workflows will further improve warehouse design and coverage of patient populations (31, 32).

Within this trial we further showed that the personalized vaccine product is well-tolerated without relevant toxicity. The most common side effects were injection side reactions, which never exceeded grade 1. Apart from a busy vaccination schedule including 16 vaccinations, treatment was easy to implement, feasible in an outpatient setting, and placed only a low burden of effort and side-effects on the patients. Concerning overall survival and disease control, no disadvantage compared to other populations of CLL patients receiving immuno-chemotherapy could be detected, as a mortality rate of 4% and median disease free survival of MRD negative patients of 26 (95% CI 23.2, 28.8) months were observed in this trial (7). Because of the small number of cases, however, these results are only of limited informative value.

It must be clearly stated that the vaccination did not achieve the expected immunogenicity outcome, as only few patients showed vaccine-induced T cell responses (see **Figure 5**). In line with other warehouse-based and “off-the-shelf” vaccine approaches (33, 34), only CLL-associated peptides with proven immunogenicity were included in the warehouse allowing for induction of T cell responses per se. This is further underlined by previously reported spontaneous memory T cell responses in CLL patients for the majority of these peptides (16), excluding peptide selection as the underlying cause for the insufficient T cell responses. T cell responses were analyzed by ELISpots after 12d *in-vitro* expansion of PBMCs with no further sorting or analyses of T cells. Due to the limited PBMC numbers, intracellular cytokine staining (ICS) could not be performed. Therefore, it is not clear whether a vaccine-induced T cell response is mediated by CD4^+^ or CD8^+^ T cells, which limits the interpretation of this study. In general CD8^+^ T cell responses are expected to be associated with HLA class I-presented peptides, while CD4^+^ responses are anticipated to be associated with HLA class II-presented peptides. Other combinations are also possible in lower frequencies due to cross-presentation and additionally direct activation of CD8^+^ T cells by embedded HLA class I T-cell epitopes. CLL patients suffer from both immunodeficiency related to the leukemia itself (humoral and cellular immune dysfunction) and as a result of CLL treatment. Within the screening process, a positive immune response to an EBV/CMV peptide mix was assessed. However, this neglected the influence of the following CLL chemoimmunotherapy, of which lymphodepletion and alterations in the T cell compartment are typical side effects (35). Prior to vaccination, CD4^+^ and CD8^+^ T cell counts were below the limit of 400/µl CD4^+^ cells in most cases, persisting throughout the vaccination period for up to 18 months. Thus, treatment-associated immunosuppression may constitute a major cause for the weak immune response to vaccination, requiring novel combinatorial approaches of standard CLL treatment and therapeutic vaccination.

As mentioned above, the treatment landscape of CLL has significantly changed since the conception of the trial reported here. First-line chemo-immunotherapy has been replaced by targeted therapies like bcl2- or BTK inhibitors (28, 36). A significant advantage of these therapies is that they do not impair the patients’ immune system, especially with regard to the T cell compartment. In fact, several studies have proven that ibrutinib as first-in-class approved BTK inhibitor does not impair T cell function and may even mediate positive effects on the T cell response. For example, it was shown that in CLL patients ibrutinib can enhance the T cell repertoire diversity (37), improve antigen presentation and reduce PD1 and PDL1 expression (38) and improve T cell numbers and function (39). In mouse models, ibrutinib improved the effect of various T cell based immunotherapies including CAR T cells, immune checkpoint inhibitors and TLR ligands (40–42). Consequently, CLL patients receiving BTK inhibitor therapy have become an even more attractive target for T cell based immunotherapies, whereas classic chemoimmunotherapy regimes like the ones the patients in our study received, have considerably lost their clinical relevance.

In this trial, topically applied imiquimod was used as an adjuvant, which induces cytokines and shows efficacy against viral infections (43). Imiquimod was used as adjuvant for peptide vaccination in several completed trials, showing the development of a proinflammatory milieu at the vaccination site (43, 44). Admittedly, single agent imiquimod never initiated particularly strong immune responses or the prevention of peptide degradation essential to achieve long lasting depot effects (45, 46). We decided therefore to employ an additional systemic adjuvant and included systemic low-dose lenalidomide for patients with MRD levels ≥ 10^-4^ in peripheral blood or bone marrow, as multiple mechanisms of action pointed to potential benefits of this combination. Lenalidomide enhances NK cell and monocyte-mediated antibody-dependent cellular cytotoxicity (47), inhibits the proliferation and function of regulatory T cells (48), and increases functional CD8^+^ and CD4^+^ T cells in CLL patients (49). Moreover, effects like an enhanced cross-priming of naïve CD8^+^ T cells by dendritic cells (50), upregulation of CD80 on tumor cells correlating with T cell activation (51) and a reversal of impaired immunological synapse formation of T cells in CLL patients (52) make lenalidomide a promising adjuvant to improve peptide-specific T cell induction. However, due to safety concerns arising from other trials in which an involvement of lenalidomide in the development of ALL could not be excluded (18, 19), lenalidomide had to be terminated, leaving the adjuvating function to locally applied imiquimod alone, which obviously did not suffice to induce potent and long-lasting T cell responses. Thus, an inability of CLL patients to mount strong immune responses and the lack of potent adjuvant formulations reasonably explains the low immunogenicity results of this trial. With regard to sample size calculation and the two step Simon design of the clinical trial, no conclusion can be made, as the required number of patients, for the first stage (n=15) was not reached in any of the arms. However, the criterion for stage two could, in theory, have been reached had there been 15 patients in each study arm (53, 54).

To overcome the latter, we recently developed and evaluated the novel toll like receptor (TLR) 1/2 agonist XS15, a water-soluble synthetic Pam_3_-Cys-derivate covalently linked to a single synthetic—nonvaccine—peptide (GDPKHPKSF) (55). XS15-adjuvanted peptide vaccines have been evaluated so far in five clinical Phase I and Phase II trials (NCT04546841 (56), NCT04954469 (57), NCT04688385 (58), NCT04842513, NCT05937295), which documented the induction of a strong CD8^+^ and Th1 CD4^+^ T cell response against vaccine peptides after subcutaneous injection in healthy volunteers as well as in cancer patients. Strikingly, the induced immune responses persisted for more than 3 years and by far exceeded T cell responses induced by other peptide vaccines as well as by mRNA vaccination (56, 57, 59, 60), suggesting XS15 as a potent novel adjuvant for peptide-based vaccine development. In view of the scarcity of T cell responses elicited in our study presented here, it has to be stated that imiquimod alone seems inadequate as adjuvant, that a combination with lenalidomide is no longer an option due to possible side effects, and that an alternative needs to be evaluated. Considering the positive results of studies employing XS15 as adjuvant, this appears to be the most promising candidate, which also appears not to require any further combinatory agents.

Both issues of limited immunogenicity and lacking clinical effectivity are addressed within a follow-up trial of our group (NCT04688385 (58). This trial applies our immunopeptidome-guided warehouse-based vaccine design to evaluate a personalized XS15-adjuvanted multi-peptide vaccine in CLL patient under BTKi treatment, with the aim to translate the lessons learned from this trial to a clinically meaningful therapeutic concept. It will employ the warehouse concept that has proven its feasibility in this trial and combine it with a potent adjuvant formulation, while pushing the therapeutical set up into the current setting of BTKi treatment.

## Data Availability

The mass spectrometry data have been deposited in the ProteomeXchange Consortium database [https://www.proteomexchange.org/] via the PRIDE partner repository (61) under dataset identifier PXD054248.
