# Supplementary material for: Warehouse-based, immunopeptidome-guided design of personalised peptide vaccines shows feasibility in clinical trial evaluation in CLL patients
Source: Front Immunol. 2024 Nov 26;15:1482715. doi: 10.3389/fimmu.2024.1482715 (PMC11628388; doi:10.3389/fimmu.2024.1482715)
Supplement: Supplementary file 1 [file DataSheet1.pdf]

Supplementary Information to the Manuscript Entitled

Warehouse-based, immunopeptidome-guided design of personalised peptide vaccines shows feasibility in clinical trial evaluation in CLL patients

Table of Contents

Supplementary Methods **2**

List of trial sites 2

Detailed inclusion and exclusion criteria for trial participants 2

HLA peptide isolation 3

Analysis of HLA ligands by liquid chromatography-coupled tandem mass spectrometry (LC-MS/MS) 3

Data processing 4

Statistical considerations regarding sample size 5

IFN-γ ELISPOT assay following 12-day in vitro expansion 6

Software and statistical analysis 7

Supplementary Tables **8**

Supplementary Table S1: *Treatment-emergent adverse events grouped by MRD status* 8

Supplementary Table S2: *Treatment-related adverse events grouped by MRD status* 12

Supplementary Table S3: *Remission status at end of study* 13

Supplementary Table S4: *Survival after vaccination* 14

Supplementary Table S5*: Reasons for treatment/study follow-up discontinuation prior to last study visit*..........15

Supplementary Table S7: *Immunopeptidome-based identification and application in vaccine cocktails of warehouse peptides* 19

Supplementary Figures: **21**

Supplementary Figure S1: Use of a high-sensitive mass spectrometer improves the detection frequency.........22

Supplementary Figure S2: Exemplary ELISpot. 23

Supplementary Figure S3: CD4 and CD8 T cell counts during vaccination 24

Supplementary Methods

List of trial sites

The trial was conducted at the Department of Hematology, Oncology, Rheumatology and Clinical Immunology, University Hospital Tübingen; the Clinical Collaboration Unit (CCU) Translational Immunology, University Hospital Tübingen; the Department of Hematology, Oncology and Palliative Care of the Katharinenhospital Stuttgart; the Department of Oncology, Hematology and Palliative Care of the Marienhospital Stuttgart; and the Department of Hematology, Oncology and Palliative Care of the Robert-Bosch-Krankenhaus Stuttgart. The Department of Hematology, Oncology, Stem Cell Transplantation and Palliative Care of the Diakonie-Klinikum Stuttgart was approved as further trial site but did not include any patients.

Detailed inclusion and exclusion criteria for trial participants

Inclusion criteria: documented diagnosis of CLL/SLL according ro IWCLL guidelines; for screening phase: no pretreatment of CLL/SLL, ability to mount an immune response (as measured by positive immune response to EBV/CMV peptide mix analyzed in 12 day recall IFNγ ELISPOT); for vaccination phase: achievement of response (at least PR according to IWCLL guidelines) after first-line therapy according to treating physician's choice; HLA typing positive for HLA alleles of peptides included in the warehouse with proven immunogenicity (HLA-A*01, A*02, A*03, A*24, B*07, B*08); ability to understand and voluntarily sign an informed consent form; age $\geq$ 18 years at the time of signing the informed consent form; ability to adhere to the study visit schedule and other protocol requirements; Eastern Cooperative Oncology Group (ECOG) performance status score of $\leq$ 2; negative serological hepatitis B and C test or negative PCR in case of positive serological test without evidence of an active infection, negative HIV test within 6 weeks prior to randomization.

Exclusion criteria: high risk CLL/SLL (17p-deletion or TP53 mutation); pregnant or lactating females; participation in any clinical study or having taken any investigational therapy, which

would interfere with the study's primary end point; patients who have received any other vaccine within 1 month prior to study inclusion; prior history of malignancies, other than CLL, unless the subject has been free of the disease for ≥ 5 years (exceptions include the following: basal cell carcinoma of the skin, carcinoma in situ of the cervix, carcinoma in situ of the breast, incidental histological finding of prostate cancer (TNM stage of T1a or T1b); disease transformation (active) (i.e. Richter's Syndrome, prolymphocytic leukemia); any immunosupressive treatment except corticosteroids.

HLA peptide isolation

HLA class I and HLA class II molecules of study patients' PBMC samples fulfilling the threshold cell count of ≥ $1x10^9$ cells were isolated (Supplementary Table S6) by standard immunoaffinity purification (1) using the pan-HLA class I-specific monoclonal antibody (mAb) W6/32, the pan-HLA class II-specific mAb Tü-39, and the HLA-DR-specific mAb L243 (all produced in-house) to extract HLA-presented peptides.

Analysis of HLA ligands by liquid chromatography-coupled tandem mass spectrometry (LC-MS/MS)

Peptide samples were measured in triplicates with shares of 20% by separating samples by reversed-phase liquid chromatography (nanoUHPLC, UltiMate 3000 RSLCnano, Thermo Fisher, Waltham, MA, USA) and subsequent analysis in an online coupled Orbitrap Fusion Lumos mass spectrometer (Thermo Fisher). Peptide separation was performed at 50 °C and a flow rate of 300 nL/min on a 50 µm × 25 cm separation column (PepMap C18, Thermo Fisher) applying a gradient ranging from 2.4% to 32.0% of acetonitrile (ACN) over the course of 90 min. Eluting peptides were ionized by nanospray ionization and analyzed in the mass spectrometer using a data-dependent acquisition mode (DDA). Remeasurements after end of study were performed in triplicates (spare cell pellets were used for a repetition of completely new HLA peptide isolation if available) or duplicates (remaining 40% shares of previously

mentioned immunopeptidome analysis for peptide cocktail design). Peptide separation was performed on Bruker Daltonic's nanoElute LC system using an acclaim TM PepMap (Thermo Fisher Scientific) and a 75 µm x 25 cm Aurora Series emitter column (IonOpticks, Fitzroy, Australia). Peptides were separated along a gradient ranging from 0% to 95% Solvent B (AcN with 0.01% FA) over the course of 60 min with consecutive ramps from 0% to 32% (30 min) and 32% to 40% (15 min), followed by two 5 min ramps to 60% and 95%, respectively, and were subsequently analyzed in the online-coupled trapped ion mobility spectrometry and time-of-flight mass spectrometer timsTOF Pro (Bruker Daltonics, Billerica, USA) equipped with a CaptiveSpray ion source using DDA(2).

Data processing

Data processing for Lumos data was performed as described previously (3). The Proteome Discoverer (v1.4, Thermo Fisher) was used for integration of search results from the SequestHT search engine (University of Washington (4)) against the human proteome (Swiss-Prot database, 20,279 reviewed protein sequences, September 27, 2013). 5% false-discovery rate (FDR) for HLA class-I and 1% FDR for HLA class II-restricted peptides were used. HLA class I binder prediction was performed using SYFPEITHI 1.0 (5) and NetMHCpan 4.1 (6). Data processing for timsTOF data was performed using the computational platform Fragpipe (v21.1) including MS Fragger (v4.0), Philosopher and MSBooster and a 5% FDR for both, HLA class I and II (7-10). Since HLA class II molecules, in contrast to HLA class I molecules, have smaller steric restrictions peptides of different lengths can fit in the binding cleft of HLA molecules. Therefore, length variants of the HLA class II-restricted peptides were determined by analyzing all result lists. Peptides were screened for either a shorter variant within the entire original peptide sequence or peptides that overlapped at least 50% with the original peptide sequence.

If one of the length variants was identified, the corresponding HLA-class II-restricted warehouse peptide was counted as identified.

Statistical considerations regarding sample size

The sample size calculation (n = 56 patients, n = 28 per arm) of the trial was based on the following assumptions: According to Simon a two-step minimax design was chosen for the statistical evaluation of the primary endpoint. We decided that if the vaccination can produce an immune response for 35% of the analyzed patients in a Phase II trial, then it is worthwhile to test this method in a large Phase III trial (acceptance of the treatment). However, if the immune response rate is no better than 20%, then the new treatment should be rejected from further development (rejection of the treatment). We decided to use α of 0.2, and power of 0.8 in such a study, using the minimax model (computed with the Software "Optimal Two-Stage Designs" by Steven Piantadosi, Cedars-Sinai Medical Center 2011, https://risccweb.csmc.edu/biostats/). Sample size calculation was done separately for each arm.

- Stage 1. $n_1 = 15$, $r_1 = 3$

    1. If not more than 3 patients have immune response (success ≤ 3) in the first 15 cases of each study arm, the treatment is abandoned (treatment rejection) at the end of stage 1. End of study

    2. With the fourth patient with immune response (success>3) anytime within the first 15 cases of each study arm, the trial enters the second stage

- Stage 2. $n_{Tot} = 28$, $r_{Tot} = 8$

Including the data from stage 1, if there are 8 or fewer patients with immune response (successes ≤ 8) at the end of 28 cases in each study arm, the treatment is abandoned (treatment rejection) as not worthy of further development: End of study. On the 9th patient

with immune response (> 8 successes) any time before 28 cases of each study arm are studied, the treatment is declared worthy of further development.

The study was terminated early due to insufficient recruitment of less than 15 patients per study arm. Therefore, the requirements for the assessment of stage 1 of the minimax design were not met and no evaluation according to the Simon Design, i.e. no interim analysis of the immune reponse was performed.

IFN-γ ELISpot assay following 12-day *in vitro* expansion

The 12-day IVE of peptide-specific T cells was performed to enable detection of low-frequent, vaccine-induced and pre-existing CLL-specific T cells. PBMCs were pulsed with vaccine-cocktail peptides (1 µg/ml and 5 µg/ml per HLA class I- and per HLA class II-restricted peptide, respectively). Irrelevant peptides with the respective HLA restrictions were used as negative controls for the expansion: GSEELRSLY for HLA-A*01 (source protein: POL_HV1H2), YLLPAIVHI for HLA-A*02 (source protein: DDX5_HUMAN), RLRPGGKKK for HLA-A*03 (source protein: GAG_HV1BR), KYPENFFLL for HLA-A*24 (source protein: PP1G_HUMAN), TPGPGVRYPL for HLA-B*07 (source protein: NEF_HV1BR), DIAARNVL for HLA-B*08 (source protein: FAK1_HUMAN), and ETVITVDTKAAGKGK for HLA class II (source protein: FLNA_HUMAN). Cells were cultured for 12 days adding 20 U/ml IL-2 (Novartis, Basel, Switzerland) on days 3, 5 and 7.

IFN-γ ELISpot assays were conducted as described before (11-13). In brief, PBMCs were stimulated with 1 µg/ml of HLA class I- or 2.5 µg/ml of HLA class II-restricted peptides and analysed in technical replicates when possible. T cell responses were considered positive if the mean spot count normalized to 500.000 cells was at least 10 spots and threefold higher than the mean spot count of the negative control. PBMCs for ELISpots were used without considering

the variable percentage of CLL populations as the maximal percentage at baseline was of 0.5% of all cells and therefore neglectable for the experiments.

Software and statistical analysis

Flow cytometric data was analyzed using FlowJo 10.7.1 (BD). Graphs were plotted using Inkscape 1.1 and GraphPad Prism 9.2.0. Statistical analyses were conducted using GraphPad Prism 9.2.0 and SAS Version 9.4.

Supplementary Tables

Supplementary Table S1: Treatment-emergent adverse events grouped by MRD status

		MRD positive (n=14)						MRD negative (n=12)				
**SOC**	**CTCAE term**	**Grade 1**	**Grade 2**	**Grade 3**	**Grade 4**	**Grade 5**	**Any grade**	**Grade 1**	**Grade 2**	**Grade 3**	**Grade 4**	**Any grade**
**Patients with events**	All terms	70	15	17	3	1	106	57	35	24	7	123
**Blood and lymphatic system disorders**	Anemia, n (%)	1 (7.1%)					1 (7.1%)	1 (8.3%)		1 (8.3%)		2 (16.7%)
	Febrile neutropenia, n (%)									1 (8.3%)†		1 (8.3%)
**Cardiac disorder**	Bradycardia, n (%)	1 (7.1%)					1 (7.1%)					
	Acute coronary syndrome, n (%)			1 (7.1%)†			1 (7.1%)					
	Aortic valve disease, n (%)									1 (8.3%)		1 (8.3%)
	Other, coronary heart disease, n (%)			2 (14.3%)†			2 (14.3%)					
	Sudden death (NOS), n (%)					1 (7.1%)†						
**Ear and labyrinth disorders**	Other, sudden hearing loss, n (%)								1 (8.3%)			1 (8.3%)
	Tinnitus, n (%)	1 (7.1%)					1 (7.1%)					
**Endocrine disorders**	Hyperparathyreodism, n (%)								1 (8.3%)†			1 (8.3%)
	Hypothyroidism, n (%)		1 (7.1%)				1 (7.1%)					
**Eye disorders**	Glaucoma, n (%)	1 (7.1%)					1 (7.1%)					
	Optic nerve disorder left, n (%)									1 (8.3%)†*		1 (8.3%)
	Optic nerve disorder right, n (%)								1 (8.3%)†*			1 (8.3%)
	Other, homonymous hemianopsia on the right eye, n (%)								1 (8.3%)			1 (8.3%)
	Other, hordeolum left eye, n (%)								1 (8.3%)			1 (8.3%)
**Gastrointestinal disorders**	Abdominal pain, n (%)							1 (8.3%)				1 (8.3%)
	Colonic perforation, n (%)				1 (7.1%)		1 (7.1%)†					
	Diarrhea, n (%)	1 (7.1%)					1 (7.1%)	1 (8.3%)		1 (8.3%)†		2 (16.7%)
	Gastroesophageal reflux disease, n (%)								1 (8.3%)			1 (8.3%)
	Gingival pain, n (%)								1 (8.3%)			1 (8.3%)
	Ileostoma closure, n (%)			1 (7.1%)†			1 (7.1%)					
	Mucositis oral, n (%)	1 (7.1%)					1 (7.1%)					
	Nausea, n (%)	1 (7.1%)					1 (7.1%)	1 (8.3%)				1 (8.3%)

	Other, lactose intolerance, n (%)							1 (8.3%)				1 (8.3%)
	Other, oral thrush, n (%)								1 (8.3%)			1 (8.3%)
	Parotis swelling, n (%)							1 (8.3%)				1 (8.3%)
	Toothache, n (%)	2 (14.3%)					2 (14.3%)					
**General disorders and administration site conditions**	Chills, n (%)	1 (7.1%)					1 (7.1%)	1 (8.3%)				1 (8.3%)
	Fatigue, n (%)	2 (14.3%)		1 (7.1%)			3 (21.4%)	1 (8.3%)				1 (8.3%)
	Fever, n (%)							1 (8.3%)				1 (8.3%)
	Flu like symptoms, n (%)	3 (21.4%)	3 (21.4%)				6 (42.9%)	5 (41.7%)				5 (41.7%)
	Injection site reaction, n (%)	11 (78.6%)					11 (78.6%)	8 (66.7%)	1 (8.3%)			9 (34.6%)
	Insomnia, n (%)							1 (8.3%)	1 (8.3%)			2 (16.7%)
	Malaise, n (%)			1 (7.1%)			1 (7.1%)	1 (8.3%)				1 (8.3%)
	Other, dysesthesia injection site, n (%)	1 (7.1%)					1 (7.1%)					
	Other, pain injection site, n (%)	1 (7.1%)					1 (7.1%)					
	Syncope, n (%)	1 (7.1%)					1 (7.1%)					
**Hepatobiliary disorders**	Other, hepatic fibrosis, n (%)		1 (7.1%)				1 (7.1%)					
**Immune system disorders**	Other, bihilar lymphadenopathy, n (%)									1 (8.3%)†		1 (8.3%)
	Other, sarcoidosis, n (%)								1 (8.3%)			1 (8.3%)
**Infections and infestations**	Lung infection, n (%)			1 (7.1%)			1 (7.1%)		1 (8.3%)			1 (8.3%)
	Nail infection, n (%)							1 (8.3%)	1 (8.3%)			2 (16.7%)
	Other, herpes genitalis infection, n (%)								1 (8.3%)			1 (8.3%)
	Other, herpes simplex labialis, n (%)							1 (8.3%)				1 (8.3%)
	Other, infection nose / throat, n (%)							1 (8.3%)				1 (8.3%)
	Other, nasolacrimal duct left eye infection, n (%)							1 (8.3%)				1 (8.3%)
	Other, shingles anal, n (%)								1 (8.3%)			1 (8.3%)
	Other, shingles, n (%)	1 (7.1%)					1 (7.1%)		1 (8.3%)			1 (8.3%)
	Sepsis, n (%)										1 (8.3%)†	1 (8.3%)
	Sinusitis, n (%)							1 (8.3%)				1 (8.3%)
	Soft tissue infection right middle finger, n (%)			1 (7.1%)			1 (7.1%)					
**Injury, poisoning and procedural complications**	Bruising, n (%)							1 (8.3%)				1 (8.3%)
	Fracture - right lower leg, n (%)			1 (7.1%)†			1 (7.1%)					
**Investigations**	Alanine aminotransferase increased, n (%)	1 (7.1%)					1 (7.1%)			1 (8.3%)		1 (8.3%)
	Aspartate, n (%)	1 (7.1%)					1 (7.1%)					

	CRP increased, n (%)		1 (7.1%)				1 (7.1%)	1 (8.3%)				1 (8.3%)
	GGT increased, n (%)									1 (8.3%)		1 (8.3%)
	Hypokalemia, n (%)							1 (8.3%)				1 (8.3%)
	LDH increased, n (%)	1 (7.1%)					1 (7.1%)	1 (8.3%)				1 (8.3%)
	Lymphocyte count decreased, n (%)	2 (14.3%)		2 (14.3%)	1 (7.1%)		5 (35.7%)			7 (58.3%)	1 (8.3%)	8 (66.7%)
	Neutrophil count decreased, n (%)		1 (7.1%)	2 (14.3%)*	1 (7.1%)		4 (28.6%)	1 (8.3%)	1 (8.3%)	2 (16.7%)	3 (25.0%)	7 (58.3%)
	Other, ß2 microglobulin increased, n(%)	1 (7.1%)					1 (7.1%)					
	Other, hypogammaglobulinemia - IgA, n (%)							1 (8.3%)				1 (8.3%)
	Other, hypogammaglobulinemia - IgG, n (%)	1 (7.1%)					1 (7.1%)	2 (16.7%)	1 (8.3%)			3 (25%)
	Other, hypogammaglobulinemia - IgM, n (%)	1 (7.1%)					1 (7.1%)	2 (16.7%)				2 (16.7%)
	Other, IgM increased, n (%)	1 (7.1%)					1 (7.1%)					
	Platelet count decreased, n (%)	3 (21.4%)					3 (21.4%)	1 (8.3%)	1 (8.3%)	1 (8.3%)		3 (25%)
	White blood cell count decreased, n (%)	1 (7.1%)	1 (7.1%)	2 (14.3%)			4 (28.6%)	1 (8.3%)	2 (16.7%)	2 (16.7%)	2 (16.7%)	7 (58.3%)
**Metabolism and nutrition disorders**	Hypercalcemia, n (%)								1 (8.3%)			1 (8.3%)
	Hyperglycemia, n (%)		1 (7.1%)				1 (7.1%)		2 (16.7%)			2 (16.7%)
	Hyperkalemia, n (%)	2 (14.3%)					2 (14.3%)					
	Hyperuricemia, n (%)	1 (7.1%)	1 (7.1%)				2 (14.3%)			1 (8.3%)		1 (8.3%)
	Hypophosphatemia, n (%)									1 (8.3%)		1 (8.3%)
**Musculoskeletal and connective tissue disorders**	Arthralgia, n (%)							1 (8.3%)				1 (8.3%)
	Back pain, n (%)	1 (7.1%)					1 (7.1%)	1 (8.3%)				1 (8.3%)
	Neck pain, n (%)	1 (7.1%)					1 (7.1%)					
	Non-cardiac chest pain, n (%)									1 (8.3%)		1 (8.3%)
	Other, arthritic complaints (left toe), n (%)								1 (8.3%)			1 (8.3%)
	Other, cervical spine pain, n (%)								1 (8.3%)			1 (8.3%)
	Other, cramp in the hand, n (%)	1 (7.1%)					1 (7.1%)					
	Other, cramps, n (%)							1 (8.3%)				1 (8.3%)
	Other, groin pain, n (%)	1 (7.1%)					1 (7.1%)					
	Other, limb pain, n (%)							1 (8.3%)				1 (8.3%)
	Other, lumbal pain, n (%)								1 (8.3%)			1 (8.3%)
	Other, omarthalgia right, n (%)							1 (8.3%)				1 (8.3%)
	Other, pain feet, n (%)								1 (8.3%)			1 (8.3%)
	Other, pain right knee, n (%)	1 (7.1%)					1 (7.1%)					

	Other, tendiniosis calcarea (left shoulder), n (%)								1 (8.3%)			1 (8.3%)
**Neoplasms benign, malignant and unspecified (incl. cysts and polyps)**	Other, basalioma left ear, n (%)	1 (7.1%)					1 (7.1%)					
	Other, parathyroidadenoma upper left, n (%)								1 (8.3%)			1 (8.3%)
**Nervous system disorders**	Dizziness, n (%)	1 (7.1%)					1 (7.1%)	2 (16.7%)	1 (8.3%)			3 (25%)
	Headache, n (%)		1 (7.1%)				1 (7.1%)	1 (8.3%)				1 (8.3%)
**Renal and urinary disorders**	Erectile dysfunction, n (%)	1 (7.1%)					1 (7.1%)					
	Other, pain left kidney, n (%)	1 (7.1%)					1 (7.1%)					
**Respiratory, thoracic and mediastinal disorders**	Cough, n (%)	1 (7.1%)					1 (7.1%)	2 (16.7%)				1 (8.3%)
	Epistaxis, n (%)							1 (8.3%)				1 (8.3%)
	Other, pulmonary nodule, n (%)	1 (7.1%)					1 (7.1%)					
	Other, subpleural fibrosis bilateral, n (%)							1 (8.3%)				1 (8.3%)
	Pleural effusion, n (%)			1 (7.1%)†			1 (7.1%)					
	Sore throat, n (%)	1 (7.1%)	1 (7.1%)				2 (14.3%)	2 (16.7%)				2 (16.7%)
	Upper respiratory infection, n (%)							1 (8.3%)				1 (8.3%)
**Nervous system disorders**	Other, lip herpes, n (%)	1 (7.1%)					1 (7.1%)					
	Other, morphea, n (%)							1 (8.3%)				1 (8.3%)
	Other, redness left shin, n (%)	1 (7.1%)					1 (7.1%)					
	Other, redness of the left middle finger, n (%)	1 (7.1%)					1 (7.1%)					
	Other, redness right shin, n (%)	1 (7.1%)					1 (7.1%)					
	Other, skin irritation perianal intermittent, n (%)	1 (7.1%)					1 (7.1%)					
	Rash acneiform, n (%)	1 (7.1%)					1 (7.1%)					
	Rash maculo-papular, n (%)	2 (14.3%)					2 (14.3%)					
	Urticaria, n (%)	1 (7.1%)					1 (7.1%)					
**Surgical and medical procedures**	Other, injury left hand, n (%)								1 (8.3%)			1 (8.3%)
**Vascular disorders**	Hematoma, n (%)	1 (7.1%)					1 (7.1%)					
	Hot flashes, n (%)	1 (7.1%)					1 (7.1%)					
	Hypertension, n (%)		3 (21.4%)	1 (7.1%)			4 (28.6%)		3 (25.0%)	1 (8.3%)		4 (33.3%)

Adverse events (AEs) and serious AEs† are classified according to CTCAE V4·03. Severity and relationship were judged by the investigator. Three events* attributed to lenalidomid were observed (one event of grade 3 neutrophile count decreased). AEs are reported until the secondary safety endpoint. Patient population was grouped according to MRD^+ and MRD^- cases. For each patient the adverse event occurring at least once was counted with the highest CTCAE grading. CTCAE, Common Terminology Criteria for Adverse Events; n, number; SOC, system organ class; CRP, C-reactive protein; γGT, γ-Glutamyltransferasen; LDH, Lactatdehydrogenase.

Supplementary Table S2: Treatment-related adverse events grouped by MRD status

		MRD positive (n=14)					MRD negative (n=12)				
**System organ class**	**CTCAE term**	**Grade 1**	**Grade 2**	**Grade 3**	**Grade 4**	**Any grade**	**Grade 1**	**Grade 2**	**Grade 3**	**Grade 4**	**Any grade**
**Gastrointestinal disorders**	Parotis swelling, n (%)						1 (8.3%)				1 (8.3%)
**General disorders and administration site conditions**	Chills, n (%)	1 (7.1%)				1 (7.1%)	1 (8.3%)				1 (8.3%)
	Fatigue, n (%)				1 (7.1%)	1 (7.1%)	1 (8.3%)				1 (8.3%)
	Fever, n (%)						1 (8.3%)				1 (8.3%)
	Injection site reaction, n (%)	11 (78.6%)				11 (78.6%)	9 (75%)				9 (75%)
	Insomnia, n (%)							1 (8.3%)			1 (8.3%)
	Other, dysesthesia injection site, n (%)	1 (7.1%)				1 (7.1%)					
	Other, pain injection site, n (%)	1 (7.1%)				1 (7.1%)					
**Investigations**	Alanine aminotransferase increased, n (%)	1 (7.1%)				1 (7.1%)					
	Aspartate aminotransferase increased, n (%)	1 (7.1%)				1 (7.1%)					
	Neutrophil count decreased, n (%)			1 (7.1%)*		1 (7.1%)	2 (16.7%)		1 (8.3%)	1 (8.3%)	4 (33.3%)
	White blood cell count decreased, n (%)								1 (8.3%)		1 (8.3%)
**Musculoskeletal and connective tissue disorders**	Arthralgia, n (%)						1 (8.3%)				1 (8.3%)
**Nervous system disorders**	Dizziness, n (%)						1 (8.3%)				1 (8.3%)
	Headache, n (%)						1 (8.3%)				1 (8.3%)
**Skin and subcutaneous tissue disorders**	Rash maculo-papular, n (%)	1 (7.1%)				1 (7.1%)					
	Urticaria, n (%)	1 (7.1%)				1 (7.1%)					
**Vascular disorders**	Hematoma, n (%)	1 (7.1%)				1 (7.1%)					

Adverse events (AEs) and serious AEs† are classified according to CTCAE V4·03. Severity and relationship were judged by the investigator. One event* also attributed to lenalidomid was observed. AEs are reported until the secondary safety endpoint. Patient population was grouped according to MRD^+ and MRD^- cases. For each patient the adverse event occurring at least once was counted with the highest CTCAE grading. CTCAE, Common Terminology Criteria for Adverse Events; n, number; SOC, system organ class.

Supplementary Table S3: Remission status at end of study

**Response at EOT**		**All (n = 26)**	**MRD positive (n = 14)**	**MRD negative (n = 12)**
Disease progression, n (%)	progressive disease	3 (11.5%)	3 (21.4%)	-
	death	1 (3.8%)	1 (7.1%)	-
MRD reduction rate[¥], n (%)	PB - CLL cell population			-
	BM - CLL cell population	2(8%)	2 (14.3%)	-
MRD negativity rate, n (%)		10 (38.5%)	-	10 (83.3%)
Missing MRD data at EOT, n (%)	PB - CLL cell population	2 (8%)	2 (14.3%)	10 (83.3%)
	BM - CLL cell population	3 (11.5%)	3 (21.4%)	11 (91.6%)

MRD assessment was routinely performed during study treatment and end of study. [¥]MRD reduction is defined as any reduction from baseline (prior to first vaccination) until last assessment; BM, bone marrow; CLL, chronic lymphocytic leukemia; EOT, end of treatment; MRD, measurable residual disease; n, number; PB, peripheral blood.

Supplementary Table S4: Survival after vaccination

		All<br>(n = 26)	MRD positive<br>(n = 14)	MRD negative<br>(n = 12)
Disease free survival				
	Patients with event, n (%)	6 (23.1%)	4 (28.6%)	2 (16.7%)
	Median, months	-	-	26
	95% CI, months	-	-	(23.2, 28.8)
Overall survival				
	Patients with event, n (%)	1 (3.9%)	1 (7.1%)	0

Disease free survival was calculated from enrollment until disease progression defined as either alternative treatment, treatment indication according to iwCLL guidelines or any cause death. Overall survival was calculated from enrollment until death from any cause. 95% CIs for survival probabilities and median survival were calculated based on a log-log transform of the survival function estimate; CI, confidence interval.

Supplementary Table S5: Reasons for treatment/study follow-up discontinuation prior to last study visit

Reasons	n (%)
Discontinuation	4 (15%)
Progressive disease	2 (8%)
Alternative treatment	-
Death	1 (4%)
AEs related to study drug	-
Lost to follow up	-
Withdrawal of consent	1 (4%)

Progressive disease is defined as CLL-associated symptoms requiring treatment according to iwCLL guidelines. AE, adverse event; n, number.

Supplementary Table S6: Patient-specific information on LC-MS/MS-based immunopeptidome analysis

Patient study number	No. of PBMCs used [x10^9]	ALC [cells/µl]	HLA type class I	Matching HLA	LC-MS/MS	follow-up	HLA class I yields	HLA class I binders	HLA class II yields	class I ware-house peptides (Lumos)	class II ware-house peptides (Lumos)	class I ware-house peptides (timsTOF)	class II ware-house peptides (timsTOF)
**UPN01**	2.20	50,490	A*02:01; B*15:01; B*56:01; C*03:04; C*01:02	A*02:01	yes	yes	8,340	7,293	7,029	3	1[1]	3	1[1]
**UPN02**	1.77	59,040	A*24:02; A*26:01; B*27:05; B*39:01; C*01:02; C*07:02	A*24:02	yes	yes	3,206	3,014	4,097	3	1[0]	5	2[0]
**UPN03**	1.00	NA	A*02; B*07; B*18; C*03; C*06	A*02; B*07	yes	yes	6,151	5,731	2,581	8	0[0]	7	0[0]
**UPN04**	2.60	51,7230	A*02; A*11; B*35; B*40; C*04; C*03	A*02	yes	yes	8,815	8,327	7,398	2	1[0]	3	1[0]
**UPN05**	1.00	33,900	A*02; A*68; B*38; B*51; C*12; C*14	A*02	yes	yes	7,117	6,178	6,760	2	1[1]	4	2[1]
**UPN06**	1.40	72,200	A*02; A*26; B*07; B*40; C*07; C*03	A*02; B*07	yes	yes	2,551	2,448	2,569	0	1[0]	NA	NA[NA]
**UPN07**	2.20	71,020	A*01; A*26; B*07; B*40; C*07; C*02	A*01; B*07	yes	yes	3,151	2,930	2,602	2	1[0]	4	0[0]
**UPN08**	2.00	19,880	A*02; A*11; B*35; B*40; C*04; C*03	A*02	yes	no	3,101	1,930	3,312	0	2[2]	2	2[2]
**UPN09**	1.00	42,6550	A*02; A*24; B*15; B*44; C*03; C*16	A*02; A*24	yes	yes	3,517	3,287	3,470	3	1[1]	8	1[0]
**UPN10**	2.30	27,900	A*02; A*11; B*40; B*44; C*03; C*05	A*02	yes	yes	3,101	2,972	4,252	0	2[2]	2	2[2]
**UPN11**	2.40	219,200	A*24; A*25; B*18; B*49; C*07; C*12	A*24	yes	yes	2,049	1,934	8,921	1	1[1]	5	0[0]
**UPN12**	1.55	192,360	A*03; A*24; B*51; C*14; C*16	A*03; A*24	yes	yes	1,213	1,050	1,261	1	1[1]	8	2[2]

**UPN13**	2.10	72,730	A*03; A*24; B*35; C*04	A*03; A*24	yes	yes	4,709	4,015	8,951	5	2[2]	9	2[0]
**UPN14**	1.80	136,130	A*02; A*24; B*07; B*44; C*07; C*05	A*02; A*24; B*07	yes	yes	9,029	8,979	10,411	9	1[0]	10	1[0]
**UPN15**	1.00	363,550	A*02; A*24; B*13; B*44; C*05; C*06	A*02; A*24	yes	no	8,154	7,655	7,028	8	1[0]	9	0[0]
**UPN16**	2.70	119,420	A*02; A*11; B*07; B*15; C*07; C*03	A*02; B*07	yes	no	7,793	7,524	7,016	4	2[1]	2	0[0]
**UPN17**	3.00	243,070	A*02; A*24; B*07; B*13; C*07; C*06	A*02; A*24; B*07	yes	yes	4,804	4,551	4,283	11	1[1]	10	0[0]
**UPN18**	2.60	245,410	A*01; A*68; B*44; B*55; C*05; C*03	A*01	yes	yes	2,841	2,695	4,522	4	1[1]	4	1[1]
**UPN19**	2.80	52,400	A*02; B*08; B*40; C*07; C*03	A*02; B*08	yes	yes	2,939	2,875	4,493	5	2[2]	7	1[1]
**UPN20**	1.90	NA	A*01; A*24; B*15; B*40; C*07; C*02	A*01; A*24	yes	yes	5,666	5,283	5,670	8	3[3]	9	2[0]
**UPN21**	2.60	127,340	A*01; A*24; B*08; B*35; C*04; C*07	A*01; A*24; B*08	yes	yes	3,756	3,606	7,270	14	2[1]	13	0[1]
**UPN22**	1.00	24,530	A*02; A*24; B*07; B*51; C*07; C*01	A*02; A*24; B*07	yes	yes	4,525	4,426	2,463	5	1[1]	9	1[1]
**UPN23**	3.00	186,890	A*01; A*26; B*08; B*38; C*07; C*12	A*01; B*08	yes	yes	3,004	2,885	4,048	3	2[1]	6	2[1]
**UPN24**	2.00	273,240	A*01; A*02; B*13; B*44; C*06; C*16	A*01; A*02	yes	yes	6,139	5,906	5,252	8	1[0]	8	1[0]
**UPN25**	1.80	87,060	A*01; A*03; B*08; B*35; C*04; C*07	A*01; A*03; B*08	yes	yes	2,586	2,479	2,872	8	2[2]	NA	NA[NA]
**UPN26**	NA	700	A*02; B*38; B*51; C*12; C*15	A*02	no	yes	NA	NA	NA	NA	NA[NA]	NA	NA[NA]

Comprehensive study patient details, including patient study number, peripheral blood mononuclear cell (PBMC) cell count (in 10^9 cells) for immunopeptidome, absolute lymphocyte count (ALC in cells/µl) and human leukocyte antigen (HLA) and warehouse matching HLA. The availability of data from tandem mass spectrometry (LC-MS/MS) immunopeptidomics and follow-up is indicated. HLA class I and HLA class II yields refer to the identified immunopeptidomics-derived peptides, HLA class I binders are predicted to bind to one of the patient's HLA class I alleles. The number of warehouse peptides identified by mass spectrometry in the patient's immunopeptidome is represented by the number of HLA class I and HLA class II warehouse peptides in either the original, pre vaccination aqcuired measurements (Lumos) or the after end of study-acquired

measurements (timsTOF). HLA class II warehouse peptides and respective length variants are represented, while detection of the exact sequence is represented in brackets. If one of the length variants was identified, the corresponding HLA-class II-restricted warehouse peptide was counted as identified. Not available (NA). Number (No.).

Supplementary Table S7: Immunopeptidome-based identification and application in vaccine cocktails of warehouse peptides

HLA allotype	Sequence	Uniprot Accession	Source Protein	Position	Detected in MS (Lumos) (n = 25)	Vaccinated (n = 26)	Allotype normalized peptide frequency (study/Lumos)	Allotype normalized peptide frequency (discovery)	Detected in MS (timsTOF) (n = 23)	Allotype normalized peptide frequency (study/ timsTOF)
**A*01**	GSDFYSMHY	Q86W34	AMZ2_HUMAN	206-214	5	4	0.71	0.50	6	1.00
**A*01**	LLDEGAKLLY	Q9NX02	NALP2_HUMAN	823-832	0	1	0.00	0.33	1	0.17
**A*01**	YLDHNSFDY	Q9BXR5	TLR10_UMAN	286-294	5	4	0.71	0.50	4	0.67
**A*01**	NTDNLLTEY	Q9Y2F5	ICE1_HUMAN	46-54	7	5	1.00	0.50	5	0.83
**A*01**	FSDPNFLAH	P16885	PLCG2_HUMAN	1142-1150	4	2	0.57	0.50	5	0.83
**A*02**	AAANIIRTL	Q13972	RGRF1_HUMAN	999-1007	2	9	0.13	0.42	4	0.29
**A*02**	YLNKEIEEA	Q9Y5B0	CTDP1_HUMAN	611-619	10	12	0.67	0.37	11	0.79
**A*02**	ILDEKPVII	Q8N139	ABCA6_HUMAN	11270-1278	5	10	0.33	0.26	12	0.86
**A*02**	SVADVVRFI	Q9UHJ3	SMBT1_HUMAN	797-805	2	8	0.13	0.21	3	0.21
**A*02**	SILEDPPSI	Q9NVM9	ASUN_HUMAN	394-402	12	15	0.80	0.16	14	1.00
**A*03**	VVKPNTSSK	Q07326	PIGF_HUMAN	61-69	0	1	0.00	0.36	0	0.00
**A*03**	RSYSSVIEK	Q9Y3R5	DOP2_HUMAN	15-23	1	1	0.33	0.55	2	1.00
**A*03**	KVSAVTLAY	P15391	CD19_HUMAN	291-299	2	2	0.67	0.36	2	1.00
**A*03**	KLYPTLVIR	Q9H9T3	ELP3_HUMAN	316-324	0	0	0.00	0.36	2	1.00
**A*03**	GLDPSQRPK	Q8WVB6	CTF18_HUMAN	358-366	0	0	0.00	0.27	1	0.50
**A*24**	VYTLLTTHL	Q8N6T0	CK080_HUMAN	447-455	10	5	0.91	0.67	11	1.00
**A*24**	KYGVFEESL	Q9BYJ4	TRI34_HUMAN	407-415	9	7	0.82	0.50	11	1.00
**A*24**	IYQQNHMVL	Q9UKT9	IKZF3_HUMAN	400-408	8	7	0.73	0.50	11	1.00
**A*24**	VYHSDIPKW	P15907	SIAT1_HUMAN	261-269	5	7	0.45	0.33	11	1.00
**A*24**	AYHNSPAYL	Q969G3	SMC1_HUMAN	132-140	4	6	0.36	0.33	11	1.00
**B*07**	SPRASGSGL	Q8WUU4	ZN296_HUMAN	218-226	4	3	0.57	0.86	3	0.50
**B*07**	APRKKSPDL	Q9BT25	HAUS_HUMAN	134-142	2	3	0.29	0.57	0	0.00

**B*07**	GPKGHVSSL	Q9H9L3	I20L2_HUMAN	190-189	4	3	0.57	0.57	1	0.17
**B*07**	KPTTSHFSVL	Q96LC7	SIG10_HUMAN	191-200	1	2	0.14	0.43	1	0.17
**B*07**	KPRAPGNLTV	P24394	IL4RA_HUMAN	122-131	4	5	0.57	0.43	4	0.67
**B*08**	QSTQRSLAL	Q9Y228	T3JAM_HUMAN	302-310	2	1	0.50	0.50	2	0.67
**B*08**	DIKSKTYSL	O76064	RNF8_HUMAN	443-451	1	1	0.25	0.50	3	1.00
**B*08**	FPLQRSVSF	Q8IZS7	CLCL1_HUMAN	100-108	4	3	1.00	0.50	1	0.33
**B*08**	ISKNKHVSL	Q9HC16	ABC3G_HUMAN	299-307	3	2	0.75	1.00	3	1.00
**B*08**	DLDVKKMPL	Q9Y6F1	PARP3_HUMAN	210-218	1	1	0.25	0.67	2	0.67
**DR**	APSQPPATYSLVNKVKNKKT	Q6UWF3	SCIMP_HUMAN	99-118	16 [7]	26	0.64 [0.28]	0.17	11 [4]	0.44 [0.16]
**DR**	YVSLYHQPAAM	O60667	FAIM3_HUMAN	366-376	3 [3]	26	0.12 [0.12]	0.20	5 [2]	0.2 [0.08]
**DR**	VKKMMKDNNLVRH	P23634	AT2B4_HUMAN	437-449	5 [4]	26	0.2 [0.16]	0.20	1 [3]	0.04 [0.12]
**DR**	DTGSYRAQISTKTSAK	Q96DU3	SLAF6_HUMAN	103-118	10 [10]	26	0.4 [0.4]	0.20	7 [4]	0.28 [0.16]

The ‘vaccinated’ column indicates the number of times a particular peptide was used within a personalized vaccine cocktail, the ‘detected in MS’ (mass spectrometry/mass spectrometer) column shows the number of study patients` immunopeptidomes in which the peptide was identified depending on the MS used. HLA class II-restricted warehouse peptides and respective lengthvariants are represented, while detection of the exact sequence is represented in brackets. If one of the length variants was identified, the corresponding HLA-class II-restricted warehouse peptide was counted as identified. Allotype normalized peptide frequencies were calculated using MS dependent absolute detections and maximal possible detections of respective peptide (which is equivalent to the number of patiens with corresponding allotype). HLA, human leucocyte antigen; n, number

Supplementary Figures:

Supplementary Figure S1: Use of a high-sensitive mass spectrometer improves the detection frequency. Boxplots of allotype normalized warehouse peptide frequencies calculated from immunopeptidomes measured on an Orbitrap Fusion Lumos mass spectrometer (dark grey) or on a timsTOF Pro (light grey). Boxes represent median and 25th and 75th percentiles, whiskers are minimum to maximum. Individual normalized frequencies of warehouse-peptides are represented by circles (Lumos) or squares (timsTOF). Statistical analysis were performed using Wilcoxon matched-pairs signed rank test.

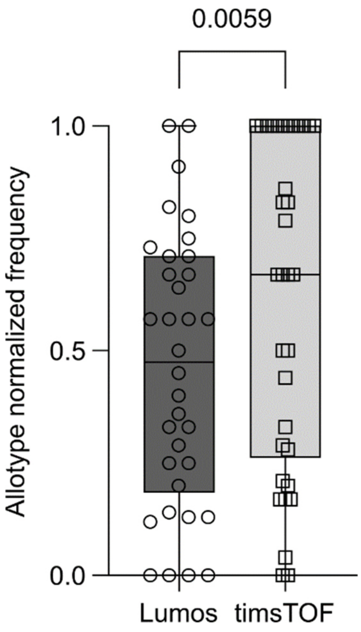

Supplementary Figure S2: Vaccine-induced T cell responses. (A) Exemplary IFN-γ T cell responses at baseline (d0) and month 10 (m10) targeting the HLA-A*02-restricted peptide SILESDPPSI compared to a negative (Neg.) control peptide (YLLPAIVHI) in patient UPN26. (B, C) Dot plots of longitudinal vaccine-induced T cell responses assessed by IFN-γ ELISpot. Data is displayed for all study patients showing a vaccine-induced T cell response to HLA class I-restricted (B) and HLA class II-restricted (C) warehouse-peptides (SILEDPPSI (SILE), VKKMMKDNNLVRH (VKKM) and APSQPPATYSLVNKVKNKKT (APSQ)) compared to negative control peptides (YLLPAIVHI or GSEELRSLY (HLA class I) and ETVITVDTKAAGKGK (HLA class II)) and positive control (Pos., phytohemagglutinin (PHA)).

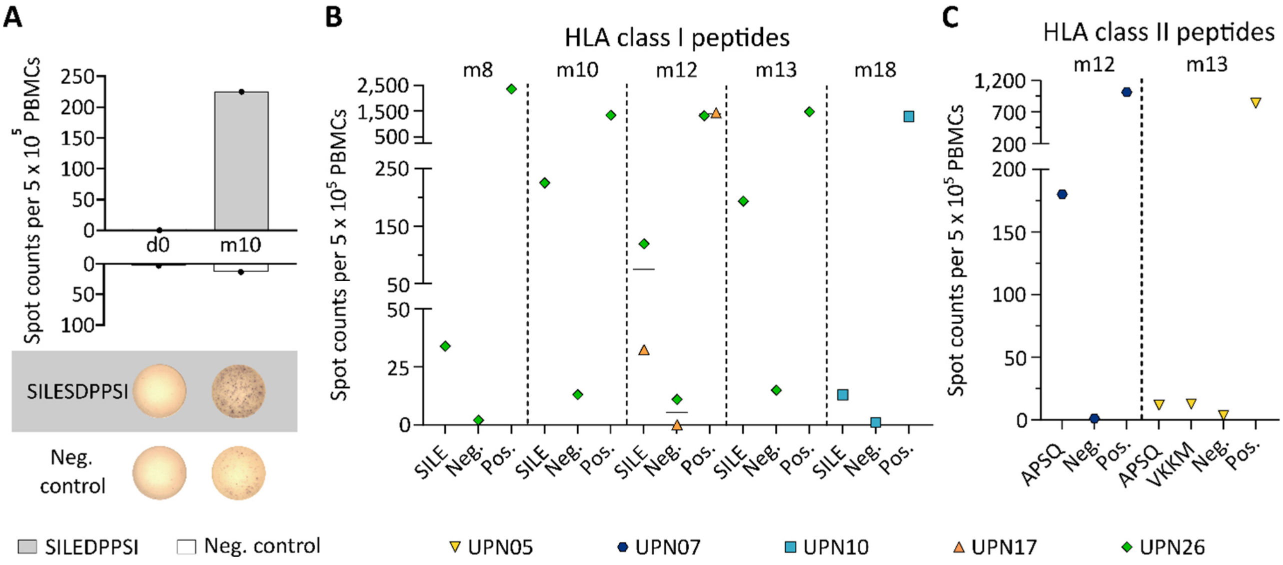

Supplementary Figure S3: CD4$^+$ and CD8$^+$ T cell counts during vaccination Immunophenotyping was performed for (left) CD4$^+$ T cell counts and (right) CD8$^+$ T cell counts at indicated time points until last follow-up.

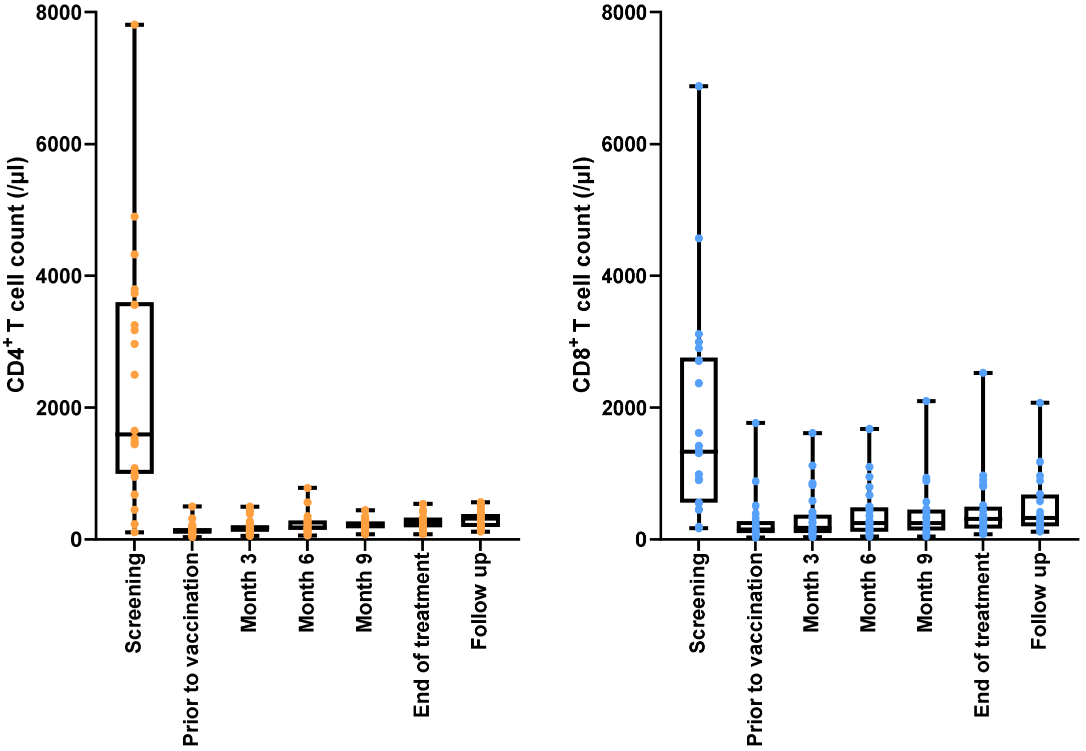

References

1. Nelde A, Kowalewski DJ, Stevanovic S. Purification and Identification of Naturally Presented MHC Class I and II Ligands. Methods Mol Biol. 2019;1988:123-36.
2. Hoenisch Gravel N, Nelde A, Bauer J, Muhlenbruch L, Schroeder SM, Neidert MC, et al. TOF(IMS) mass spectrometry-based immunopeptidomics refines tumor antigen identification. Nat Commun. 2023;14(1):7472.
3. Kowalewski DJ, Schuster H, Backert L, Berlin C, Kahn S, Kanz L, et al. HLA ligandome analysis identifies the underlying specificities of spontaneous antileukemia immune responses in chronic lymphocytic leukemia (CLL). Proc Natl Acad Sci U S A. 2015;112(2):E166-75.
4. Eng JK, McCormack AL, Yates JR. An approach to correlate tandem mass spectral data of peptides with amino acid sequences in a protein database. J Am Soc Mass Spectrom. 1994;5(11):976-89.
5. Schuler MM, Nastke MD, Stevanovikc S. SYFPEITHI: database for searching and T-cell epitope prediction. Methods Mol Biol. 2007;409:75-93.
6. Reynisson B, Alvarez B, Paul S, Peters B, Nielsen M. NetMHCpan-4.1 and NetMHCIIpan-4.0: improved predictions of MHC antigen presentation by concurrent motif deconvolution and integration of MS MHC eluted ligand data. Nucleic Acids Res. 2020;48(W1):W449-W54.
7. da Veiga Leprevost F, Haynes SE, Avtonomov DM, Chang HY, Shanmugam AK, Mellacheruvu D, et al. Philosopher: a versatile toolkit for shotgun proteomics data analysis. Nat Methods. 2020;17(9):869-70.
8. Kong AT, Leprevost FV, Avtonomov DM, Mellacheruvu D, Nesvizhskii AI. MSFragger: ultrafast and comprehensive peptide identification in mass spectrometry-based proteomics. Nat Methods. 2017;14(5):513-20.
9. Yang KL, Yu F, Teo GC, Li K, Demichev V, Ralser M, et al. MSBooster: improving peptide identification rates using deep learning-based features. Nat Commun. 2023;14(1):4539.
10. Yu F, Haynes SE, Teo GC, Avtonomov DM, Polasky DA, Nesvizhskii AI. Fast Quantitative Analysis of timsTOF PASEF Data with MSFragger and IonQuant. Mol Cell Proteomics. 2020;19(9):1575-85.
11. Heitmann JS, Bilich T, Tandler C, Nelde A, Maringer Y, Marconato M, et al. A COVID-19 peptide vaccine for the induction of SARS-CoV-2 T cell immunity. Nature. 2022;601(7894):617-22.
12. Heitmann JS, Tandler C, Marconato M, Nelde A, Habibzada T, Rittig SM, et al. Phase I/II trial of a peptide-based COVID-19 T-cell activator in patients with B-cell deficiency. Nat Commun. 2023;14(1):5032.
13. Nelde A, Maringer Y, Bilich T, Salih HR, Roerden M, Heitmann JS, et al. Immunopeptidomics-Guided Warehouse Design for Peptide-Based Immunotherapy in Chronic Lymphocytic Leukemia. Front Immunol. 2021;12:705974.
